# Integrating TSPO PET imaging and transcriptomics to unveil the role of neuroinflammation and amyloid-β deposition in Alzheimer’s disease

**DOI:** 10.1007/s00259-023-06446-3

**Published:** 2023-10-06

**Authors:** Miao Zhang, Xiao-hang Qian, Jialin Hu, Yaoyu Zhang, Xiaozhu Lin, Wangxi Hai, Kuangyu Shi, Xufeng Jiang, Yao Li, Hui-dong Tang, Biao Li

**Affiliations:** 1 Department of Nuclear Medicine, Ruijin Hospital, Shanghai Jiao Tong University School of Medicine, Shanghai, China; 2 Department of Geriatrics, Ruijin Hospital, Shanghai Jiao Tong University School of Medicine, Shanghai, China; 3 Medical Center On Aging of Ruijin Hospital, Shanghai Jiao Tong University School of Medicine, Shanghai, China; 4 Department of Neurology and Institute of Neurology, Ruijin Hospital, Shanghai Jiao Tong University School of Medicine, Shanghai, China; 5 School of Biomedical Engineering, Shanghai Jiao Tong University, Shanghai, China; 6 Department of Nuclear Medicine, Bern University Hospital, University of Bern, Bern, Switzerland; 7 Department of Informatics, Technische Universität München, Munich, Germany

**Keywords:** TSPO, [^18^F]DPA-714 PET/MR, *CD200*, Neuroinflammation, Amyloid-β, Alzheimer’s disease

## Abstract

**Purpose:**

Despite the revealed role of immunological dysfunctions in the development and progression of Alzheimer’s disease (AD) through animal and postmortem investigations, direct evidence regarding the impact of genetic factors on microglia response and amyloid-β (Aβ) deposition in AD individuals is lacking. This study aims to elucidate this mechanism by integrating transcriptomics and TSPO, Aβ PET imaging in clinical AD cohort.

**Methods:**

We analyzed 85 patients with PET/MR imaging for microglial activation (TSPO, [^18^F]DPA-714) and Aβ ([^18^F]AV-45) within the prospective Alzheimer’s Disease Immunization and Microbiota Initiative Study Cohort (ADIMIC). Immune-related differentially expressed genes (IREDGs), identified based on AlzData, were screened and verified using blood samples from ADIMIC. Correlation and mediation analyses were applied to investigate the relationships between immune-related genes expression, TSPO and Aβ PET imaging.

**Results:**

TSPO uptake increased significantly both in aMCI (*P* < 0.05) and AD participants (*P* < 0.01) and showed a positive correlation with Aβ deposition (r = 0.42, *P* < 0.001). Decreased expression of *TGFBR3, FABP3, CXCR4 and CD200* was observed in AD group. *CD200* expression was significantly negatively associated with TSPO PET uptake (r =—0.33, *P* = 0.013). Mediation analysis indicated that *CD200* acted as a significant mediator between TSPO uptake and Aβ deposition (total effect B = 1.92, *P* = 0.004) and MMSE score (total effect B =—54.01, *P* = 0.003).

**Conclusion:**

By integrating transcriptomics and TSPO PET imaging in the same clinical AD cohort, this study revealed *CD200* played an important role in regulating neuroinflammation, Aβ deposition and cognitive dysfunction.

**Supplementary Information:**

The online version contains supplementary material available at 10.1007/s00259-023-06446-3.

## Introduction

Alzheimer’s disease (AD) is the most common type of dementia, affecting more than 50 million people worldwide [1, 2]. The histopathological signature of AD is characterised by extracellular plaques containing aggregates of various amyloid-β (Aβ) peptides and intraneuronal neurofibrillary tangles containing hyperphosphorylated tau [3, 4]. Current evidence implicates multiple pathogenic processes involved in AD etiology, neuroinflammation, commonly associated with microglial reactivity, has been increasingly recognized as an important contributor to AD pathogenesis [5–7], and there is huge potential for therapies that modulate the neuro-immune or microglia response in AD [8, 9].

Microglia, astrocytes, and neurons act synchronously to promote neurodegeneration [7]. Microglia are innate immune cells of the myeloid lineage that reside in the central nervous system (CNS) [10, 11]. As an innate primary response, microglia seem to have a protective role in the presence of amyloid plaques or a pro-inflammatory, damaging role, involved in the spread of tau tangles in AD [12, 13]. Effective in vivo imaging monitoring is crucial for studying the immune pathogenesis of AD [14]. The 18-kDa translocator protein (TSPO)-positron emission tomography (PET) imaging allows the quantification of microglial and astrocytes activation and visualisation of CNS neuroinflammation in patients with AD in vivo [15, 16]. Upon microglial and astrocyte activation, TSPO levels are significantly increased [17]. High [^18^F]DPA-714 PET uptake was correlated with favourable clinical evolution [18]. Furthermore, TSPO-PET imaging of microglia activation is a key element linking the effect of Aβ to tau spread and ultimately dementia [12].

Characterisation of the genetic landscape of AD provides a unique opportunity for a better understanding of immune-associated pathophysiological processes. With the development of sequencing technology, several risk genes involved in the regulation of microglia function were identified in AD, such as *TREM2*, *CD33*, and *ABCA7* [19]. Our previous studies have shown that immunological changes were present in MCI by integrating peripheral blood and brain tissue transcriptomic analyses [20]. Animal and postmortem evidences showed genetic factors affected the microglia reaction and amyloid-β deposition [21–23]. The characterization of this association in living AD patients is critical to confirm immune mechanisms to support therapeutic strategies. However, studies in clinical AD patients are still lacking.

For the first time, we integrated brain tissue transcriptomics (based on the AlzData database), peripheral blood transcriptomics, brain TSPO- and Aβ-PET imaging in the patients from the same clinical AD cohort (ADIMIC), and explored associations between immune-related transcriptomic and brain inflammation changes. We hypothesised that transcriptomic changes would regulate neuroinflammation and Aβ deposition, subsequently influencing cognitive dysfunction in patients with AD.

## Materials and methods

### Study design and participants

All participants were prospectively recruited from Alzheimer’s Disease Immunization and Microbiota Initiative study Cohort (ADIMIC) (http://www.chictr.org.cn, identifier: ChiCTR2100046493). This study was approved by the review board of Ruijin Hospital, Shanghai Jiao Tong University School of Medicine research committee (approval number: 2021-46) and was performed in accordance with the Helsinki declaration. All participants provided written informed consent prior to participating.

We recruited 85 participants from Ruijin Hospital, Shanghai Jiao Tong University School of Medicine between May 2021 and June 2022, including 28 healthy controls (HCs), 28 patients with amnestic mild cognitive impairment (aMCI), and 29 patients with AD. All groups were matched for age and sex (Table 1). A consensus diagnosis of aMCI was made according to the Petersen criteria [24]. The clinical diagnosis of AD was made by the panel based on the NIA-AA criteria [25]. Exclusion criteria were (1) individuals with structural lesions on the brain, such as brain infarctions, haemorrhage, traumatic brain injury, and so on; (2) those with contraindications to magnetic resonance imaging MRI; (3) those with substantial reductions in serum B12, red cell folate, thyroid function levels; (4) those with autoimmune and systemic inflammatory diseases; and (5) those using corticosteroids, non-steroidal anti-inflammatory, or benzodiazepines, which interfere with TSPO imaging [18].

**Table 1:** Demographic and clinical information

Characteristic	HC	a-MCI	AD	*P* value
Number	28	28	29	/
Age, years, mean (SD)	66.1 ± 6.2	69.6 ± 4.9	66.4 ± 7.1	0.070
Sex (male: female)	11:17	14:14	12:17	0.692
Education, years, mean (SD)	12.54 ± 3.89	10.36 ± 3.96	9.62 ± 4.03	0.032
*rs6971*(HAB)	28	28	29	/
*APOE4* + , number (%)	4 (14.3%)	13 (46.4%)	16 (55.2%)	0.003
Amyloid PET positive (%)	0	16 (57.1%)	26 (89.7%)	< 0.001
SUVR of [^18^F]DPA-714 PET, mean (SD)	1.08 ± 0.56	1.14 ± 0.07	1.16 ± 0.06	< 0.001
SUVR of [^18^F] AV-45 PET, mean (SD)	0.97 ± 0.06	1.22 ± 0.27	1.38 ± 0.24	< 0.001
Mean CDR scores	0	0.5	1.69 ± 0.47	< 0.001
MMSE (mean ± SD)	29.21 ± 1.13	26.46 ± 1.53	15.83 ± 5.12	< 0.001
MoCA (mean ± SD)	26.70 ± 3.59	20.59 ± 3.51	9.84 ± 4.13	< 0.001

**Fig. 1:**
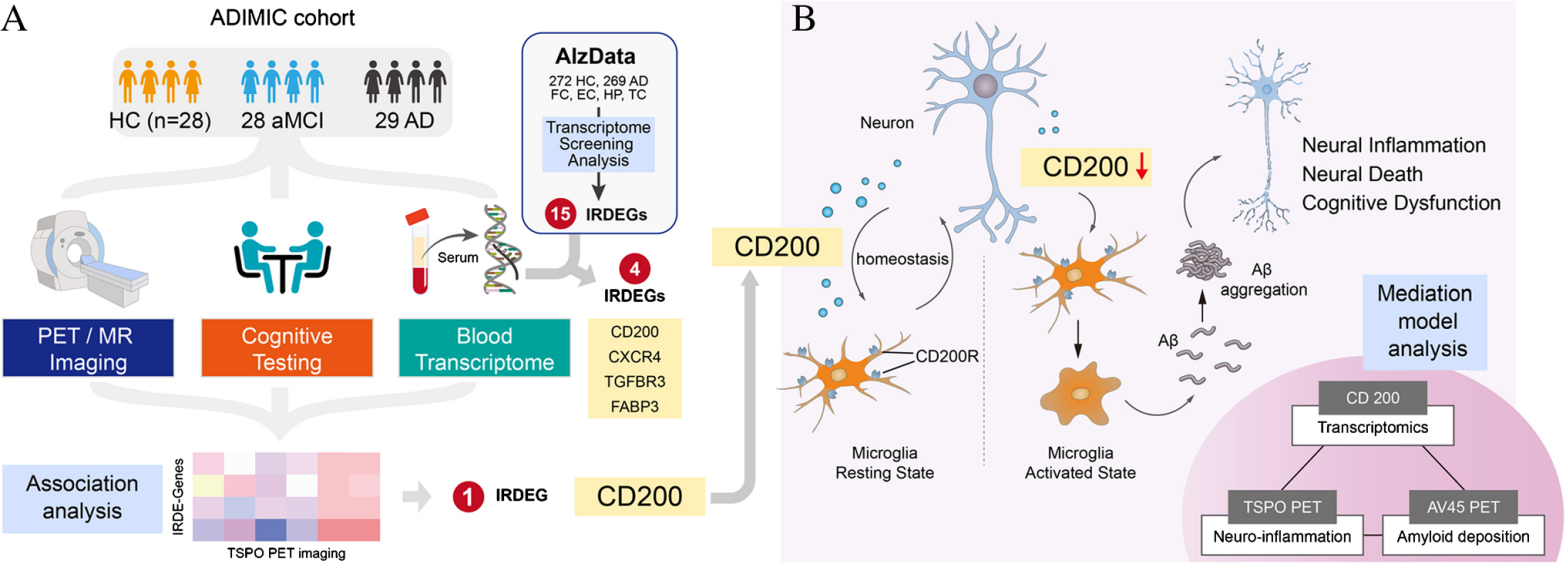
The study flowchart. **A** Eighty-five participants were enrolled from ADIMIC cohort, who underwent [^18^F]DPA-714, [^18^F]AV45 PET/MR exams and neuropsychological assessment. Based on the AlzData database, IRDEGs in post-mortem brain tissues were screened and enrichment analysis were performed. Further, we verified the expression levels of the IRDEGs in the blood of our study cohort and confirmed that the immune-related transcriptomic biomarkers *CD200* level was statistically decreased in AD patients and significantly negatively correlated with TSPO-PET uptake. **B** Under physiological conditions, neuronal-microglial crosstalk via the CD200-CD200R signalling pathways to maintain the homeostatic profiles of microglia. In AD patients, *CD200* decreased and activated microglia to aggravate Aβ deposition and neural death, cognitive dysfunction which was confirmed by mediation model

All participants underwent the same procedure including a complete clinical and neuropsychological assessment, [^18^F]DPA-714 and [^18^F]AV-45 PET/magnetic resonance (MR) scanning and blood sample collection. The study flowchart is illustrated in Fig. 1.

### Neuropsychological assessment

Each participant was assessing the Mini-Mental State Examination (MMSE, Chinese version), global clinical dementia rating (CDR), Beijing version of the Montreal Cognitive Assessment (MoCA), Rey Auditory Verbal Learning Test, Digital Span Test, Stroop Test, Animal Fluency Test, Boston Naming Task, Self-rating depression scale, and activity of daily living questionnaire.

### TSPO genotype

Blood samples were drawn to characterise TSPO genotypes. Based on the rs6971 polymorphism within the *TSPO* gene, we classified all participants as high-affinity binders (HAB), mixed-affinity binders or low-affinity binders (LAB) [15, 18]. We screened the TSPO genotypes of each participant included in this study.

### Screening of immune-related differentially expressed genes (IRDEGs) in post-mortem brain tissues and enrichment analysis

Transcriptome data of the entorhinal cortex, hippocampus, temporal cortex, and frontal cortex were downloaded from the AlzData database, which includes high-throughput omic data (genomics, transcriptomes, proteomics, and functional genomics) [26]. The IRDEGs between 272 HCs and 269 patients with AD were analysed using the ‘limma’ package in R under the screening condition of a *P*-value < 0.05 and |log_2_ fold change|> 0. A volcano plot was produced by Graphpad 9.0 to visualise the total differentially expressed genes (DEGs) between the HC and AD groups. Finally, IRDEGs were obtained by integrating DEGs and immune genes from the InnateDB (https://www.innatedb.com/) and ImmPort (https://www.immport.org/home) databases using a Venn diagram [27, 28]. Gene ontology (GO) and Kyoto Encyclopedia of Genes and Genomes (KEGG) pathways analyses were used to determine the functional roles of IRDEGs using the ‘clusterProfiler package’ in R (significance level, *P*-value < 0.05).

### Detection of RNA expression in peripheral whole blood

Peripheral whole blood was stored in a -80 °C freezer before RNA extraction after collecting blood from each participant within 30 min. RNA transcriptome sequencing was performed at the Shanghai Applied Protein Technology Co., Ltd. (APTBIO, Shanghai, China). Details of the RNA detection method are provided in Appendix E1.

### [^18^F]DPA-714 and [^18^F]AV-45 PET/MR imaging acquisition

All participants underwent [^18^F]DPA-714 and [^18^F]AV-45 PET/MR scanning within 2 weeks. PET and MRI were performed with a 3.0-T hybrid PET/MR scanner (Biograph mMR; Siemens Healthcare, Erlangen, Germany) using the vendor supplied 12-channel phase-array head coil.

#### MRI imaging

MRI was performed simultaneously with PET data acquisition. First, we performed three-dimensional T1-magnetisation prepared-rapid gradient echo (MPRAGE) and fluid-attenuated inversion recovery (FLAIR) sequences followed by axial two-dimensional T2 and susceptibility weighted imaging, coronal T2, and coronal FLAIR sequences. Coronal slices were angulated perpendicular to the hippocampal long axis.

#### PET imaging

For [^18^F]DPA-714 PET imaging, a mean dose of 260.83 ± 37.96-MBq [^18^F]DPA-714 was injected intravenously, and PET dynamic acquisitions in list mode lasted 60–90 min after injection. For [^18^F]AV-45 PET imaging, a mean dose of 260.65 ± 40.34-MBq [^18^F]AV-45 was injected intravenously, and statistic PET images were acquired at 40–60 min after the bolus injection. Both [^18^F]DPA-714 PET and [^18^F]AV-45 PET images were reconstructed using the ordered subset expectation maximisation algorithm (subsets = 21, iterations = 4) and post-filtered with an isotropic full-width half-maximum Gaussian kernel of 2 mm, 128 slices per slab, gap of 0.5 mm; and matrix size of 344 × 344.

### Visual assessment of PET/MR imaging

The MR and PET images were visually analysed by three experienced radiologists with certificates in nuclear medicine and radiology. Details of visual assessment of PET/MR imaging are provided in Appendix E1.

### PET/MR image processing

Native-space T1-weighted MRI of each participant was segmented and parcellated into cortical and subcortical regions by FreeSurfer 6.0.0 (https://surfer.nmr.mgh.harvard.edu/). Then, [^18^F]DPA-714 PET and [^18^F]AV-45 PET images were linearly co-registered to the 1-mm isotropic anatomical MRI by advanced normalisation tools. The cerebellar grey matter was used as the reference region for [^18^F]DPA-714 PET to calculate the standard uptake value ratio (SUVR) map. The whole cerebellum was used as the reference region for [^18^F] AV-45 PET to calculate SUVR map. The mean SUVR was calculated within 34 bilateral cortical regions of interest defined by the Desikan–Killiany Atlas. The volumes of interest were defined separately for the left and right hemispheres and were then pooled into greater anatomical volumes of interest. We defined 16 volumes of interest including the frontal lobe, parietal lobe, occipital lobe, temporal lobe, precuneus, entorhinal cortex, hippocampus, para-hippocampus, amygdala, anterior cingulate, posterior cingulate (PCC), thalamus, caudate, putamen, striatum, and white matter [15, 29].

### Statistical analysis

GraphPad Prism 5.0 software (GraphPad Software, Inc., San Diego, CA, USA) and Sangerbox online software (http://sangerbox.com/) were used to perform statistical analysis and graphing. Data normality was tested using the Kolmogorov–Smirnov test. Demographic and clinical characteristics were compared among the HC, aMCI, and AD groups using the chi-square test for categorical variables and one-way analysis of variance (ANOVA) for continuous variables. For the whole brain and 16 brain regions of interest, [^18^F]AV-45 and [^18^F]DPA-714 SUVRs were obtained for each participant and compared between the three groups using analysis of covariance with age, sex and education as covariates. Then, Spearman’s correlation analyses were performed to explore the correlations between the [^18^F]DPA-714 SUVR and MMSE score, MoCA score, and blood *CD200, TGFBR3, FABP3*, and *CXCR4* expression levels.

Mediation analysis is a statistical model used to quantify a mediating variable affects a dependent variable, and we performed structural equation modelling using AMOS software with a level of confidence of 95% and 5000 bootstrap samples. Mediation analysis comprised total, direct, and indirect effects. The percent of mediation (Pm) calculated by the indirect effect divided by the total effect was determined to study the weight of the *CD200* level on the total effect. Statistical significance was defined as a *P*-value <0.05.

## Results

### Participant characteristics

We enrolled 28 HCs (17 women; mean age, 66.1 years), 28 patients with aMCI (14 women; mean age, 69.6 years), and 29 patients with AD (17 women; mean age, 66.4 years). All groups were matched for sex (*P* = 0.692) and age (*P* = 0.070) (Table 1). All participants were genotyped for the TSPO polymorphism (rs6971) and identified as having TSPO HAB*.* The raw data of rs6971 polymorphism test was shown in Table S1. Among 29 patients with AD, 26 participants had positive amyloid PET imaging with a global SUVR of > 1.11. Among 28 patients with aMCI, 16 participants showed positive amyloid PET imaging.

### [^18^F]DPA-714 PET uptake was associated with Aβ deposition and cognitive dysfunction

**Fig. 2:**
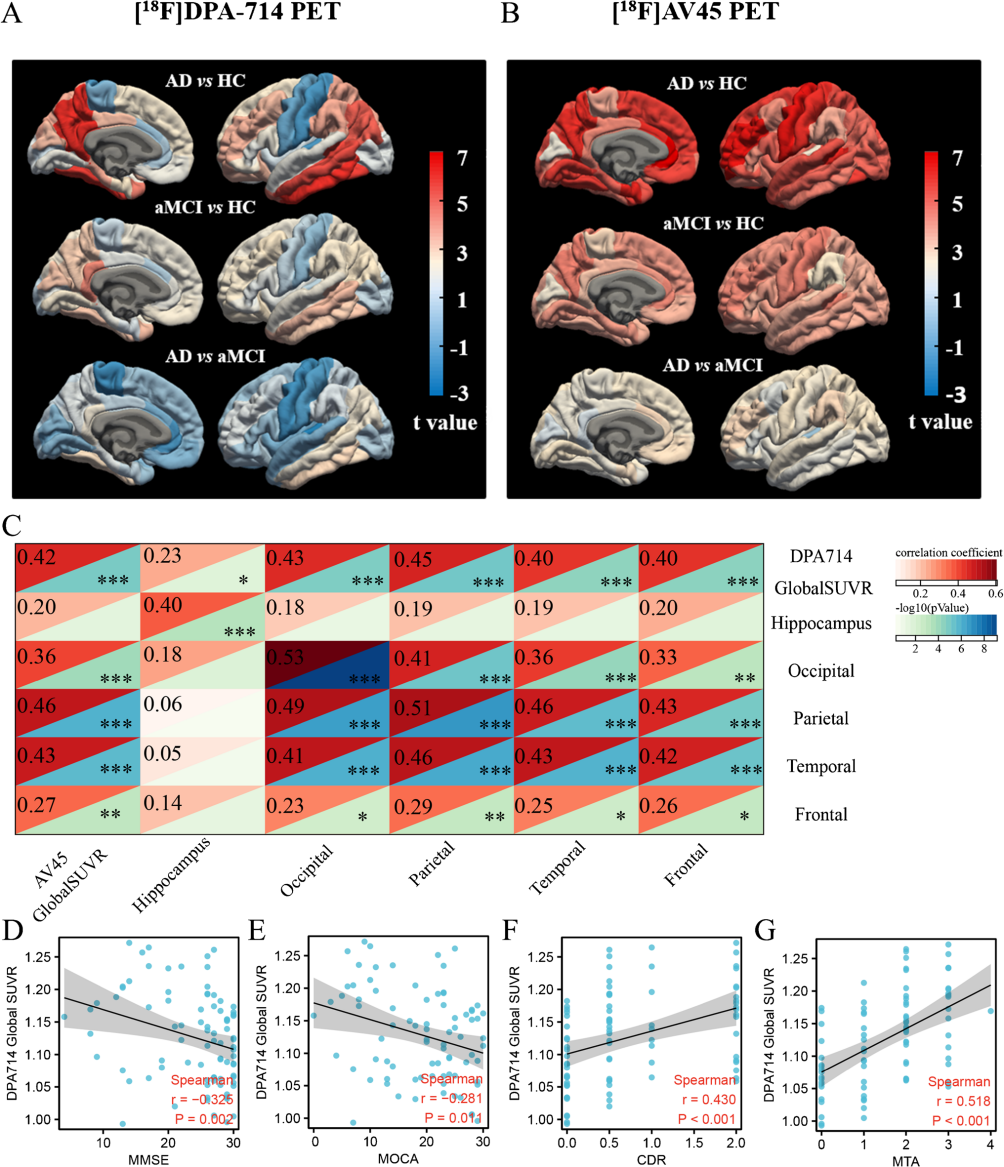
[^18^F]DPA-714 PET uptake was associated with amyloid deposition and cognitive clinical assessment. **A**, **B** Significantly higher [^18^F]DPA-714 and [^18^F]AV45 PET uptake were observed in patients with aMCI and AD compared to HCs (t-value -3 ~ 7). **C** The partial correlation matrix with age, sex and education partialled out reveals that the TSPO PET uptake is significantly correlated with amyloid burden in the global brain cortex (r = 0.42, *P* < 0.001) as well as occipital lobe, parietal lobe, temporal lobe. **D**, **E** The [^18^F]DPA-714 SUVR is negatively correlated with MMSE (r = -0.325, *P* = 0.002) and MOCA score (r = -0.281, *P* = 0.011). **F**, **G** The [^18^F]DPA-714 SUVR is positively correlated with CDR (r = 0.430, *P* < 0.001) and MTA stage, respectively (r = 0.518, *P* < 0.001)

[^18^F]DPA-714 PET and [^18^F]AV-45 PET uptakes were progressively higher in patients with aMCI and AD compared with HCs (Fig. 2A and B). Significantly increased [^18^F]DPA-714 SUVRs of the global brain cortex, frontal, temporal, occipital, partial lobe, (para)hippocampus, amygdala, PCC, precuneus, and entorhinal cortex were observed in the aMCI and AD groups (*P* < 0.05) (Table S2).

Partial correlation matrics with age, sex and education partialled out revealed that TSPO-PET uptake was significantly correlated with Aβ deposition in the global brain cortex (r = 0.42, *P* < 0.001) (Fig. 2C) and hierarchically correlated in occipital lobe (r = 0.53, *P* < 0.001), parietal lobe (r = 0.51, *P* < 0.001), temporal lobe (r = 0.43, *P* < 0.001).

The SUVR of [^18^F]DPA-714 showed a negative correlation with the MMSE (r = -0.325, *P* = 0.002) (Fig. 2D) and MoCA scores (r = -0.281, *P* = 0.011) (Fig. 2E), and a positive correlation with the CDR (r = 0.430, *P* < 0.001) (Fig. 2F) and MTA stage (r = 0.518, *P* < 0.001) (Fig. 2G).

### Screening and enrichment analysis of IRDEGs by integrating brain transcriptomic

**Fig. 3:**
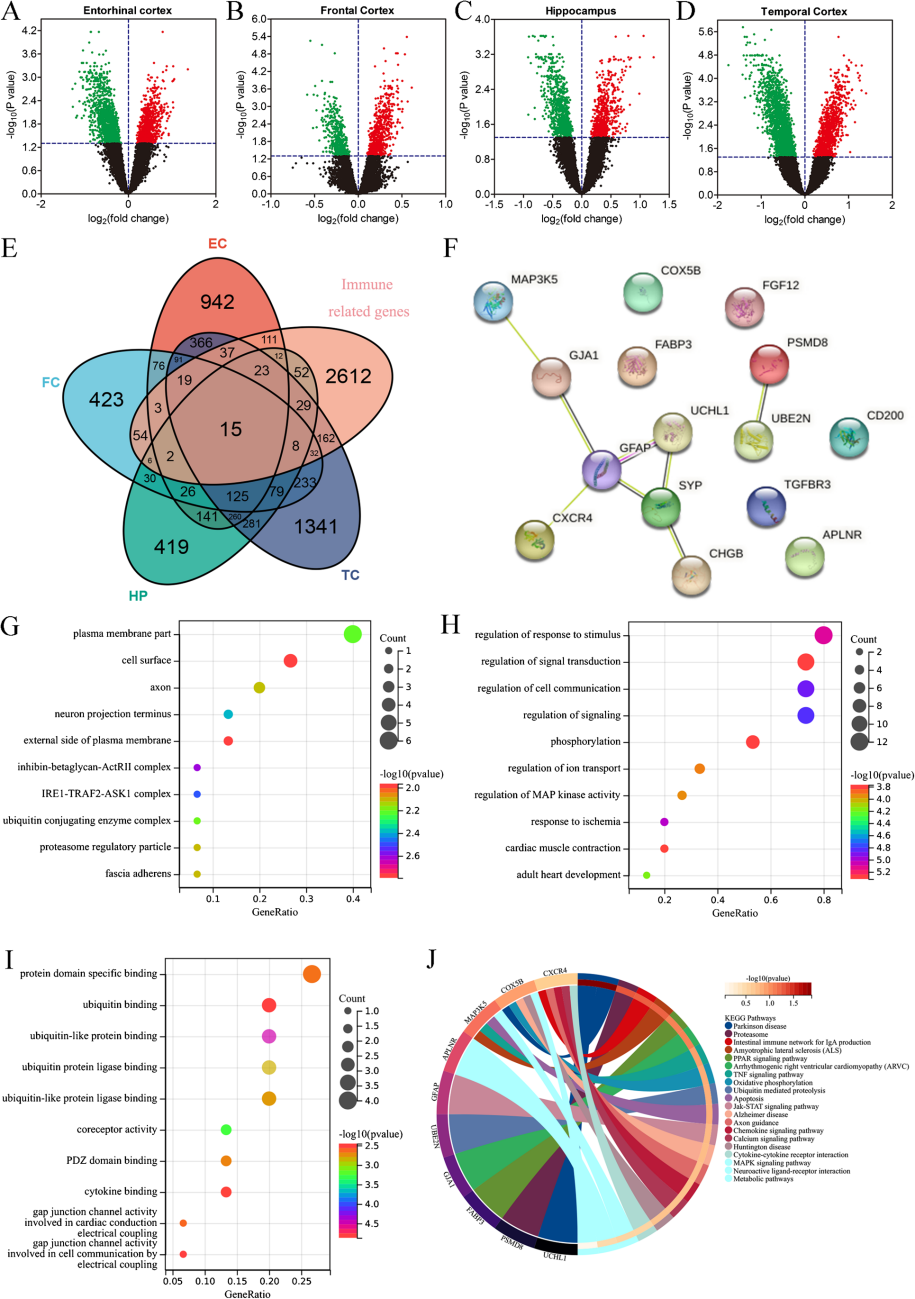
Screening of immune-related differentially expressed genes (IRDEGs) in post-mortem brain tissues and enrichment analysis. **A**–**D** Transcriptome expression data of 272 HCs and 269 AD patients from the AlzData database used to screen immune-related differentially expressed genes (IRDEGs). **E** There are 140 differentially expressed genes with common changes in four brain regions between HC and AD groups. **F** After integrating immune-related gene sets from the InnateDB and ImmPort database, we finally screened 15 IRDEGs between the HC and AD groups. **G** Cellular components analysis shows that they are located in the plasma membrane, cell surface, and axon. **H** Biological process, **I** Molecular function, **J** KEGG enrichment were analysed

To further explore the immunological characteristics of AD, we obtained the entorhinal cortex, frontal cortex, hippocampus, and temporal cortex transcriptome expression data of 272 HCs and 269 patients with AD from the AlzData database to screen IRDEGs (Fig. 3A–D). Under the aforementioned screening conditions, we screened out 140 differentially expressed genes with common changes in four brain regions between the HC and AD groups (Fig. 3E). After integrating immune-related gene sets from the InnateDB and ImmPort database, we finally screened 15 IRDEGs *(APLNR, TGFBR3, PSDM8, FABP3, CHGB, CXCR4, GFAP, FGF12, UCHL1, COX5B, UBE2N, MAP3K5, SYP, CD200,* and *GJA1*) between the HC and AD groups (Fig. 3F). Among them, the expression levels of *APLNR, TGFBR3, CXCR4, GFAP, MAP3K5,* and *GJA1* were up-regulated in the AD group, whereas those of *PSMD8, FABP3, CHGB, FGF12, UCHL1, COX5B, UBE2N, SYP,* and *CD200* were down-regulated in the AD group compared with those in the HC group (Table S3). We further speculated the molecular functions of these 15 IRDEGs using GO functional and KEGG enrichment analyses. Cellular components analysis shows that the IRDEGs were located in the plasma membrane, cell surface, and axon (Fig. 3G). Biological process analysis showed that IRDEGs were involved in regulation of the response to stimulus, signal transduction, and cell communication (Fig. 3H). Molecular function was related with protein domain-specific binding, ubiquitin binding, and coreceptor activity (Fig. 3I). KEGG enrichment analysis revealed that IRDEGs were related with AD, Parkinson’s disease, the tumour necrosis factor (TNF) signalling pathway, Jak-STAT signalling pathway, and MAPK signalling pathway (Fig. 3J).

### Immune-related gene *CD200* expression was associated with [^18^F]DPA-714 PET uptake

**Fig. 4:**
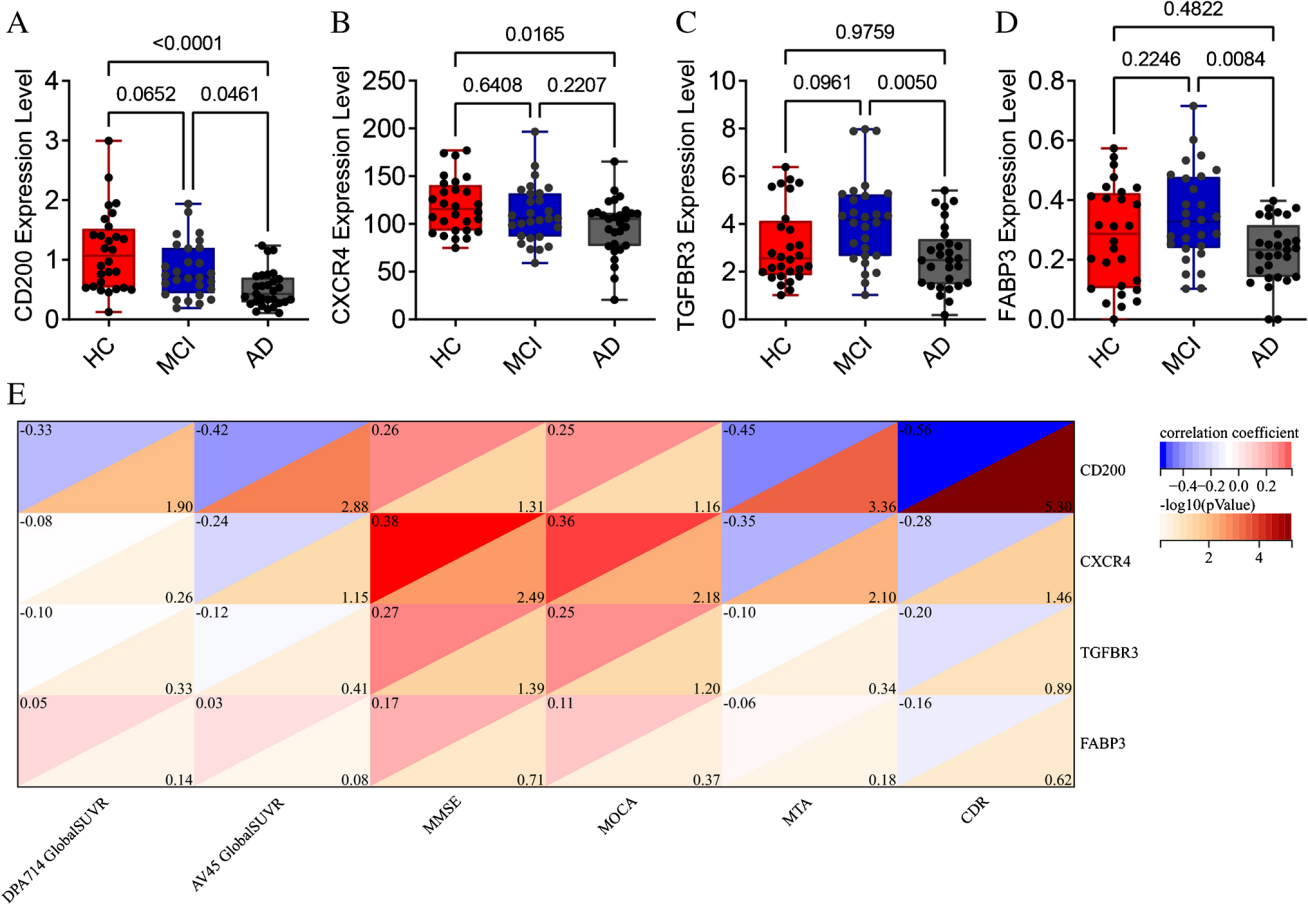
Verification of IRDEGs in blood samples from ADIMIC cohort and their correlation analysis with TSPO PET imaging. **A**–**D** The *CD200, CXCR4, TGFBR3* and *FABP3* expression level in the Alzheimer’s disease (AD) group is lower than that in HC groups. **E** The partial correlation matrix reveals that the immune-related gene *CD200* expression level is negatively associated with [^18^F]DPA-714 uptake in the global standard uptake value ratio (SUVR) in HC + AD groups (r =—0.33, *P* = 0.013). Blood *CXCR4, TGFBR3, FABP3* expression levels show no statistical correlation with the SUVR in [^18^F]DPA-714 PET imaging

We further verified the expression levels of the 15 aforementioned IRDEGs in the peripheral blood of the ADIMIC. The *CD200* expression level was lower in the AD group than in the HC and aMCI groups (Fig. 4A). There was a decreased expression level of *CXCR4* in the AD group compared with the HC group (Fig. 4B). The expression levels of *TGFBR3* and *FABP3* were decreased in the AD group compared with the aMCI group (Fig. 4C and D). In the HC and AD groups, the expression levels of *FABP3* and *CD200* in peripheral blood were consistent with that in brain tissue in the AlzData database. The remaining 11 IRDEGs did not show statistical differences between the three groups (Fig. S1). Our current data showed no statistically significant differences in the transcriptional expression of blood TREM2, CD33 and ABCA7 among HCs, individuals with MCI, and AD patients (Fig. S2).

Therefore, we analysed the correlation between the four reliably verified IRDEGs (*TGFBR3, FABP3, CXCR4,* and *CD200*) and the clinical and neuroimaging features associated with AD. Blood *CXCR4, TGFBR3, FABP3* expression levels showed no statistical correlation with the SUVR in [^18^F]DPA-714 PET imaging (Table S4 and S5).

Interestingly, our data proved that the *CD200* expression level was negatively associated with [^18^F]DPA-714 uptake in the HC and AD groups including the global SUVR (r =—0.330, *P* = 0.013) (Fig. 4E) and SUVRs of the frontal lobe, parietal lobe, temporal lobe, parahippocampal cortex, PCC, precuneus, striatum, amygdala, and white matter (*P* < 0.05) (Table S4). The *CD200* expression level was also correlation with the AV-45 global SUVR (r =—0.42, *P* < 0.01). It was positively correlated with MMSE (r = 0.26,* P* < 0.01) and MoCA scores and negatively correlated with and MTA stage (r =—0.45, *P* < 0.001) and CDR (r =—0.56, *P* < 0.001) in the HC and AD groups (Fig. 4E).

Furthermore, we examined the correlation between CD200 and SUVR of DPA-714 PET in different groups, including HC, MCI, AD, HC + MCI, HC + AD and HC + MCI + AD, respectively (Fig. S3). A significant association between blood CD200 levels and [^18^F] DPA-714 uptake was only found in the HC + AD group (r = -0.330, *P* = 0.013). In the MCI group, CD200 expression showed a positive correlation with DPA-714 PET SUVR, although it did not reach statistical significance (r = 0.344, P = 0.073). Conversely, in the AD group, CD200 levels exhibited a negative correlation with SUVR (r = -0.102, *P* = 0.599).

### *CD200* was a significant mediator between neuroinflammation, Aβ deposition, and cognitive dysfunction

**Fig. 5:**
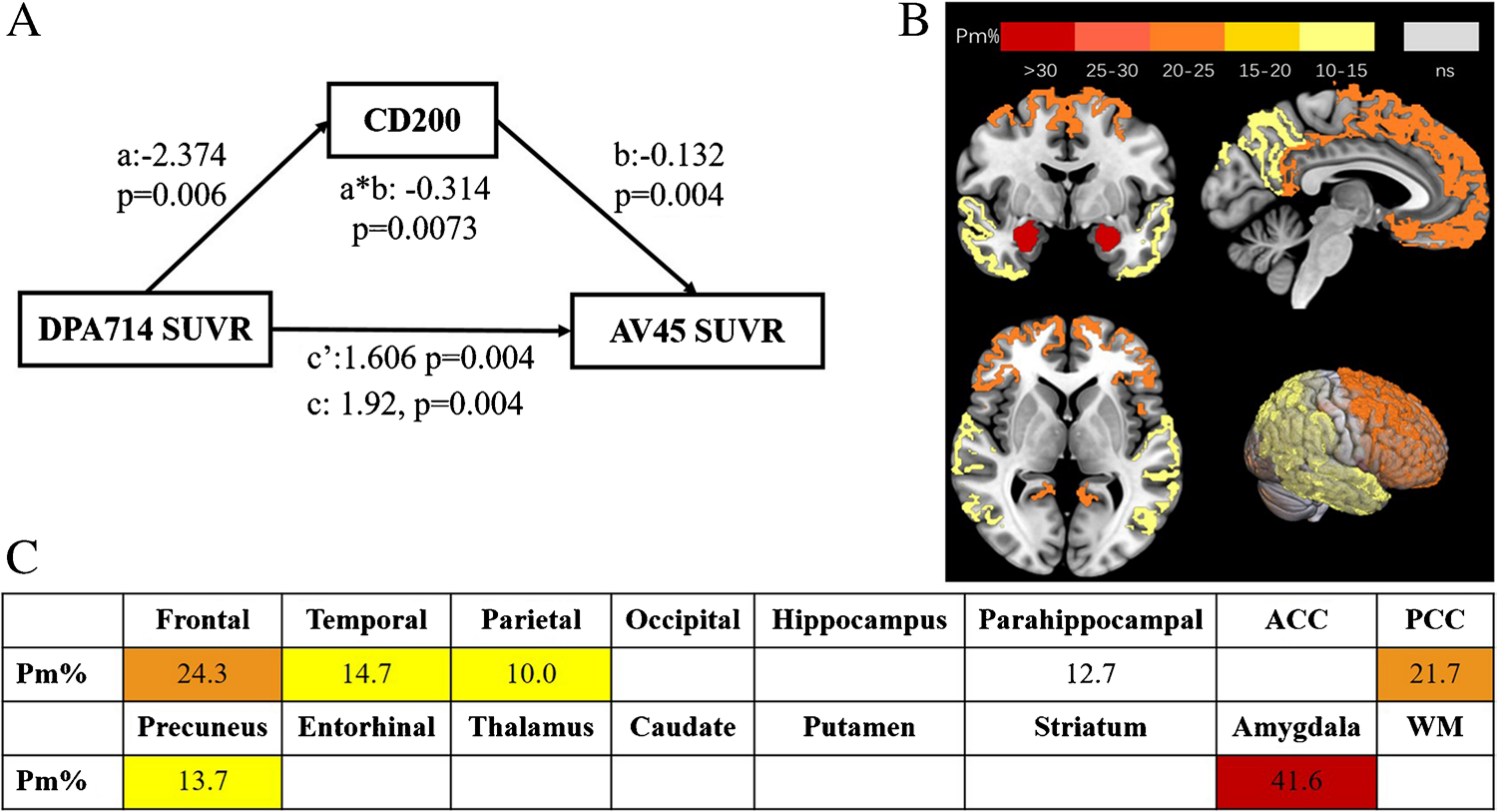
Mediation analysis of the blood *CD200* expression levels between TSPO uptake and amyloid deposition. **A** The total effects of *CD200* mediated DPA714 and AV45 PET uptake were positively significant (total effect B = 1.92, *P* = 0.004). **B** and **C** The topographical distribution showed *CD200* was a significant mediator between DPA714 uptake and AV45 uptake in the amygdala (percent of mediation [Pm] = 41.6%; *P* = 0.000), frontal lobe (Pm = 24.3%; *P* = 0.002), posterior cingulate (PCC) (Pm = 21.7%; *P* = 0.002)

In the mediation analysis of the blood *CD200* expression level with the TSPO uptake and *Aβ* deposition, the total effects were positively significant according to the global SUVR (total effect B = 1.92, *P* = 0.004, Fig. 5A). *CD200* showed significant mediation effects between the regional SUVRs of [^18^F]DPA-714 and AV-45 PET in the amygdala (Pm = 41.6%; *P* = 0.000), frontal lobe (Pm = 24.3%; *P* = 0.002), PCC (Pm = 21.7%; *P* = 0.002), parietal lobe (Pm = 10.0 %;* P* = 0.041), and precuneus (Pm = 13.7%; *P* = 0.013) (Fig. 5B and C).

**Fig. 6:**
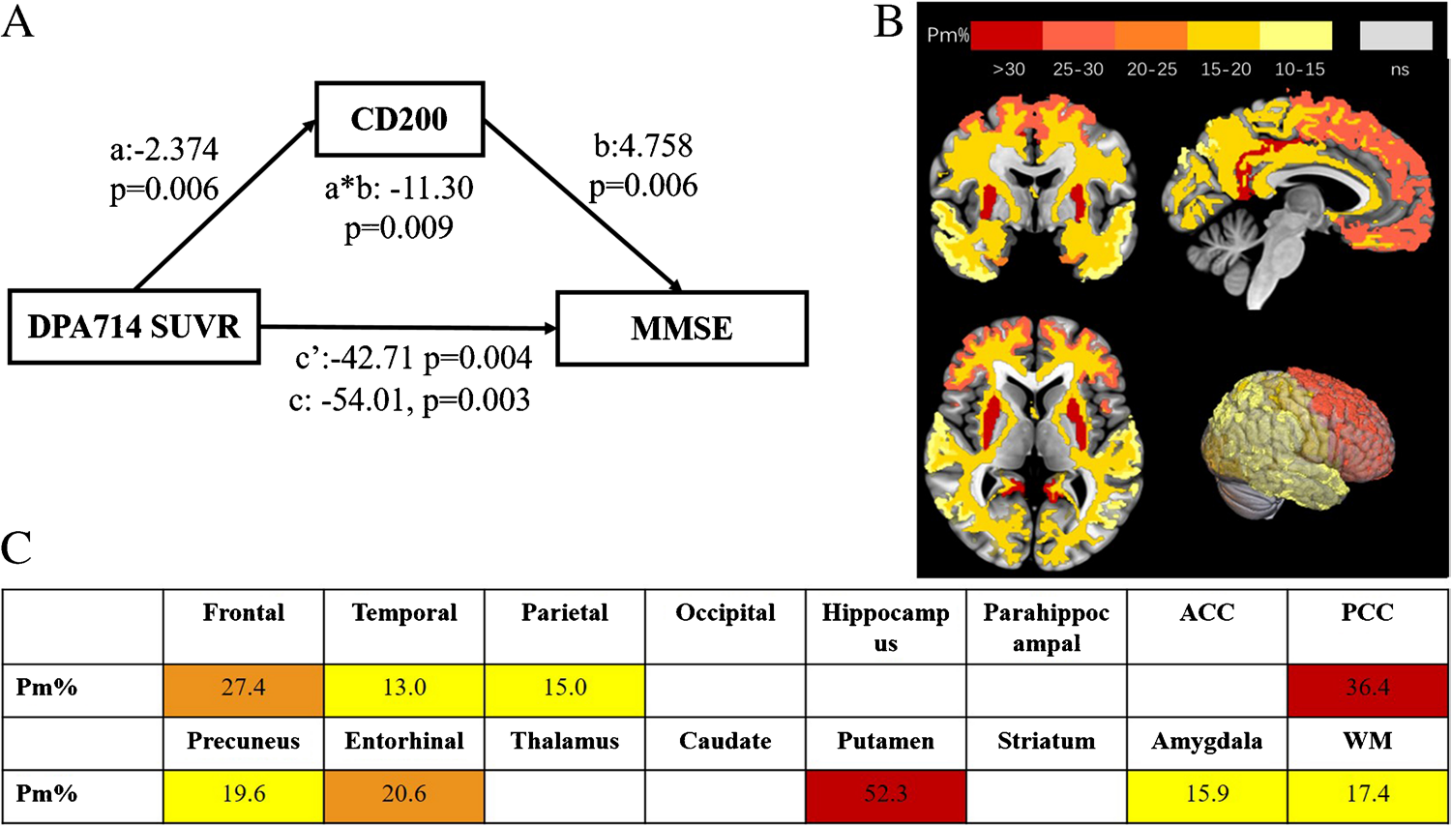
Mediation analysis of the blood *CD200* expression levels between TSPO uptake and MMSE score. **A** The total effect of *CD200* mediated DPA714 and cognitive assessment was negatively significant (total effect B = -54.01, *P* = 0.003). **B** and **C** Topographical distribution of *CD200* mediated [^18^F]DPA-714 uptake and MMSE score in putamen ([Pm] = 52.3%; *P* = 0.000), posterior cingulate (PCC) (Pm = 36.4%; *P* = 0.000), frontal, temporal, parietal lobe, and entorhinal cortex (Pm range, 13.0–52.3%)

In the mediation analysis of the blood *CD200* expression level with the TSPO uptake, cognitive dysfunction (MMSE score), the total effects were negatively significant according to the global SUVR (total effect B = -54.01, *P* = 0.003, Fig. 6A). *CD200* showed significant mediation effects between the regional SUVRs of [^18^F]DPA-714 and the MMSE scores in the putamen (Pm = 52.3%; *P* = 0.000), PCC (Pm = 36.4%; *P* = 0.000), frontal cortex, and entorhinal cortex (Pm range, 13.0–52.3%, Fig. 6B and C).

The mediator model analysis in the direction of AV45 towards DPA-714 was showed in Fig. S4. *CD200 *showed no significant mediation effect between globe SUVR of [^18^F]AV45 and [^18^F]DPA-714(indirect effect B = 0.011, *P* = 0.371).

## Discussion

### Main findings

Dysregulation of the immune system is a cardinal feature of AD [7]. The association of neuroinflammation imaging and immune-related transcriptomics can help us understand biological mechanisms behind the brain imaging phenotype and innate immune pathological change that may be amenable to pharmacological intervention [30]. Animal and postmortem studies indicated genetic factors affected the microglia reaction and amyloid-β deposition. However, studies in clinical AD patients are still lacking. Herein, for the first time, we attempted to integrate transcriptomics with PET imaging to explore immunity-related mechanics in the same clinical AD cohorts. Interestingly, our study showed that *CD200* acted as a significant mediator between PET uptake of TSPO, Aβ deposition, and cognitive dysfunction in clinical AD cohort (Fig. 1).

### [^18^F]DPA-714 PET uptake was associated with Aβ deposition

Microglia and astrocytes are the predominant mediators of inflammation within the central nervous system. TSPO is a transmembrane domain protein that has minimal expression in the brain at physiological levels [31]. Upon microglial and astrocyte activation, TSPO levels increased significantly [29].

TSPO-PET imaging is used to directly visualise neuroinflammation in vivo [14]. Novel [^18^F]fluorinated second generation TSPO radio-tracers, such as [^18^F]DPA-714, have greater affinity and a better signal-to-noise ratio than the early generation tracer [32]. Our study showed that [^18^F]DPA-714 PET uptake increased in large scale brain regions of patients with aMCI and those with progressive AD (Fig. 2). The [^18^F]DPA-714PET SUVR was correlated with Aβ deposition and cognitive scores, consistent with previous studies’ findings [15, 17, 29].

We conducted TSPO genotype screening for each participant included in this study. It’s worth noting that all participants in this cohort exhibited high-affinity binders (HABs) for TSPO, indicating that [^18^F]DPA-714 PET imaging is particularly suitable for application in Chinese or Asian populations. As previous studies showed second-generation TSPO radioligands have suffered from a high inter individual variability in binding due to sensitivity to the rs6971 polymorphism of the TSPO gene [33]. This polymorphism results in high affinity binders (HABs), low affinity binders (LABs) and mixed affinity binders (MABs) [34], whereas low-affinity binders (LABs) are unsuitable for evaluation [35]. According to the HapMap database, in American and Europe, the percentage of HABs was 69.2% and 45.7%; In East Asia, the frequency of the HAB genotype is 96.9% [36]*.*

### Immune-related genes expression was associated with TSPO uptake

Martins et al.’s study [30] proved that a novel method called ‘imaging transcriptomics’ can recover biological correlates for a range of benchmark molecular imaging markers in the healthy human brain. However, their data didn’t confirm the correlation between immune related transcriptomic and TSPO PET uptake. Their study was based on the AHBA database, which included six post-mortem healthy specimens. TSPO is upregulated during neuroinflammation, but in the healthy brain, the TSPO signal is low. Another reason was that the data of TSPO-PET imaging and transcriptomics were from different cohorts. All these limitations caused the inconclusive results of the correlation between TSPO imaging and transcriptomics expression. In the present study AlzData was used for transcriptome analysis at the brain tissue level. To mitigate the potential influence of racial disparities, the identified IRDEGs from AlzData were subsequently validated in the blood samples of participants from the ADIMIC cohort. Moreover, the correlation analysis between TSPO PET imaging and blood transcriptomics was performed within the same group of subjects from the ADIMIC cohort.

*CD200 *expression level significantly decreased in AD patients. Furthermore, the blood *CD200 *expression level was significantly associated with TSPO-PET uptake. Our current data did not exhibit statistically significant variations in the expression of *GFAP, TREM2, CD33 *and *ABCA7*, either at the post-mortem brain tissue level or at blood transcription levels, between HC and AD groups. Several factors may contribute to these findings: firstly, the inclusion of post-mortem brain tissues from the AlzData database; secondly, our utilization of peripheral blood samples instead of cerebrospinal fluid (CSF); thirdly, our focus on blood transcriptional changes rather than protein levels. It’s worth noting that our study consistently demonstrated significant results for *CD200*, both in post-mortem brain tissues from the AlzData database and in blood samples from the ADIMIC cohort. This consistent pattern suggests a pivotal role for CD200 in immune-related regulation in AD, possibly serving as a novel and effective biomarker for AD diagnosis and therapy.

CD200 is a type I membrane glycoprotein of the immunoglobulin superfamily of cell surface proteins, which presents on neurons in the rodent brain and interacts with the CD200 receptor (CD200R), which is a myeloid cell receptor found on microglia [37]. Neurons and microglia participate in dynamic bidirectional communication that is essential for brain homeostasis [7, 38]. Under physiological conditions, neuronal-microglial crosstalk via the CD200-CD200R and CX3CL1-CX3CR1 signalling pathways to maintain the homeostatic profiles of microglia [7, 10, 38]. Under chronic and acute neuroinflammatory conditions, CD200 expression is negatively correlated with microglial activation (J neurosci 2007). In this study, we observed that in the MCI group, *CD200 *expression was positively correlated with DPA-714 PET uptake. Conversely, in the AD group, *CD200 *levels exhibited a negative correlation with DPA-714 uptake. In HC or MCI participants, the CD200-CD200R signaling pathways appeared to facilitate neuronal-microglial crosstalk, maintaining the homeostatic profiles of microglia. However, in AD patients with amyloid deposition, the downregulation of CD200 disrupted this physiological balance, leading to microglial activation.

### The mediation model showed that *CD200* mediated neuroinflammation to aggravate Aβ deposition and cognitive decline in AD patients

Amyloid-β can serve as a class of molecules known as danger-associated molecular patterns, triggering the immune response. This response may subsequently be amplified by additional immunostimulatory molecules released or generated during ongoing neuroinflammation [39]. Conversely, in early stage of AD, disease-associated microglia (DAM) can form a barrier to reduce further deposition of plaques and actively participate in the disassembly and digestion of β-amyloid plaques [40]. In progressive stages of AD, microglia exhibit a pro-inflammatory phenotype, contributing to the exacerbation of Aβ deposition [7]. In the present study, mediation model analysis of patients with AD showed that peripheral *CD200* mediated neuroinflammation, Aβ deposition and cognitive decline in AD. Additionally, the association was region specific.

Previous animal studies reported that CD200 plays a vital role in neuroinflammation and Aβ deposition. Lee JH and colleagues’ study confirmed faulty autolysosome acidification in AD mouse models induced autophagic build-up of Aβ in neurons, yielding senile plaques [41]. CD200 is capable of enhancing microglia-mediated Aβ clearance and neural differentiation, holding potential as a therapeutic avenue for AD [42]. The inefficient clearance of Aβ engulfed by microglia is a major pathogenic pathway in AD, which might be attributed to increased cytokine concentrations and downregulation of Aβ phagocytosis receptor expression [43]. Consequently, in AD patients with amyloid deposition, the down-regulation of CD200 results in compromised microglial function for Aβ clearance, leading to aggravated amyloid deposition and cognitive decline.

The total effects of mediator model analysis in the opposite direction from AV45 towards DPA-714 has no statistical significance. The reason is the receptor for CD200, known as CD200R is predominantly present on the microglia instead of Aβ plaques, resulting in the specific immune related axis which maintains the CNS homeostasis.

Furthermore, PET/MR imaging in vivo also provided important new insight to visualise the neuroinflammation changes of different brain regions. We found that *CD200* mediated TSPO, Aβ uptake, and cognitive dysfunction in the PCC, precuneus, amygdala, frontal, temporal, parietal lobe, and putamen. These regions play vital roles in attention, memory, and executive function. Acting as a central hub in the interconnected brain network, the PCC and precuneus showed disrupted functional connectivity and hypometabolism [44], and preferential vulnerability to amyloid pathology in the early stage of AD [45]. Cortical hubs such as the PCC and precuneus may cause preferential vulnerability of neuroinflammation and aggravate amyloid accumulation and cognitive dysfunction.

### Limitation

This study has some limitations. Firstly, this study’s results need to be further confirmed by a larger sample cohort and more long-term observation. Secondly, animal experiments need to be performed to elucidate the biological mechanisms and cell pathways of *CD200* as a new immune-related biomarker of AD. Thirdly, it’s important to note that this study does not encompass the analysis of neuroinflammation and tau pathology, which warrants further investigation in future research endeavors.

## Conclusion

By integrating transcriptomics and TSPO PET imaging within the same clinical AD cohort, this study revealed that the immune-related gene CD200 plays an important role in regulating neuroinflammation, Aβ deposition and cognitive dysfunction. This finding holds promise CD200 as a novel and impactful biomarker for both the diagnosis and treatment of AD.

### Supplementary Information

Below is the link to the electronic supplementary material.

Supplementary file1 (DOCX 16 KB)

Supplementary file2 (PDF 1047 KB)

Supplementary file3 (JPG 1479 KB)

Supplementary file4 (JPG 376 KB)

Supplementary file5 (JPG 760 KB)

Supplementary file6 (JPG 1028 KB)

Supplementary file7 (DOCX 31 KB)

## Data Availability

Anonymized data are available upon collaborative requests. Further inquiries can be directed to the corresponding authors.
