# Supplementary material for: Integrating TSPO PET imaging and transcriptomics to unveil the role of neuroinflammation and amyloid-β deposition in Alzheimer’s disease
Source: Eur J Nucl Med Mol Imaging. 2023 Oct 6;51(2):455–67. doi: 10.1007/s00259-023-06446-3 (PMC10774172; doi:10.1007/s00259-023-06446-3)
Supplement: Supplementary file 1 — Supplementary file1 (DOCX 16 KB) [file 259_2023_6446_MOESM1_ESM.docx]

**Integrating TSPO PET imaging and transcriptomics to unveil the role of neuroinflammation and amyloid-β deposition in Alzheimer's disease**

***Appendix E1***

**Materials and methods**

**Detection of RNA expression in peripheral whole blood**

Peripheral whole blood was stored in a -80°C freezer before RNA extraction after collecting blood from each participant within 30 minutes. RNA transcriptome sequencing was performed at the Shanghai Applied Protein Technology Co., Ltd. (APTBIO, Shanghai, China). Total RNA was extracted using the TRIzol® Reagent according to the manufacturer’s instructions (Magen Biotech, Waltham, MA, USA). Next, the A260/A280 absorbance ratio and RNA integrity number were measured by Nanodrop ND-2000 (Thermo Scientific, Waltham, MA, USA) and an Agilent Bioanalyzer 4150 system (Agilent Technologies, Santa Clara, CA, USA), respectively, to assess the quality of the extracted RNA. Then, paired-end libraries were constructed using the ABclonal mRNA-seq Lib Prep Kit (ABclonal, Hubei, China) according to the manufacturer’s instructions. The library quality was assessed using an Agilent Bioanalyzer 4150 system (Agilent Technologies). The library preparations were sequenced on an Illumina Novaseq 6000 (Illumina, San Diego, CA, USA), and 150-bp paired-end reads were generated. The raw data of the fastq format were processed through in-house Perl scripts to remove the adapter sequence and filter out low-quality data, and aligned with the reference genome using the orientation mode of HISAT2 software (http://daehwankimlab.github.io/hisat2/). The reads numbers mapped to each gene were counted using FeatureCounts (<http://subread.sourceforge.net/>). Finally, the length and reads count mapped to this gene were applied to analyse the fragments per kilobase per million mapped fragments of each gene.

**Visual assessment of PET/MR imaging**

The MR and PET images were visually analysed by three experienced radiologists with certificates in nuclear medicine and radiology*.* A standardised scale (Scheltens scale) was used to rate the medial temporal lobe atrophy (MTA) from T1-MPRAGE, coronal T2, and coronal FLAIR MR images. Additionally, [^18^F]AV-45 PET images were visually assessed based on the International Nuclear Medicine Consensus on the Clinical Use of Amyloid Positron Emission Tomography in AD.(Tian et al., 2022)

**References**

Tian, M., Zuo, C., Civelek, A. C., Carrio, I., Watanabe, Y., Kang, K. W., . . . Zhang, H. (2022). International Nuclear Medicine Consensus on the Clinical Use of Amyloid Positron Emission Tomography in Alzheimer’s Disease. *Phenomics*. doi: 10.1007/s43657-022-00068-9
