# Supplementary material for: Integrating TSPO PET imaging and transcriptomics to unveil the role of neuroinflammation and amyloid-β deposition in Alzheimer’s disease
Source: Eur J Nucl Med Mol Imaging. 2023 Oct 6;51(2):455–67. doi: 10.1007/s00259-023-06446-3 (PMC10774172; doi:10.1007/s00259-023-06446-3)
Supplement: Supplementary file 2 — Supplementary file2 (PDF 1047 KB) [file 259_2023_6446_MOESM2_ESM.pdf]

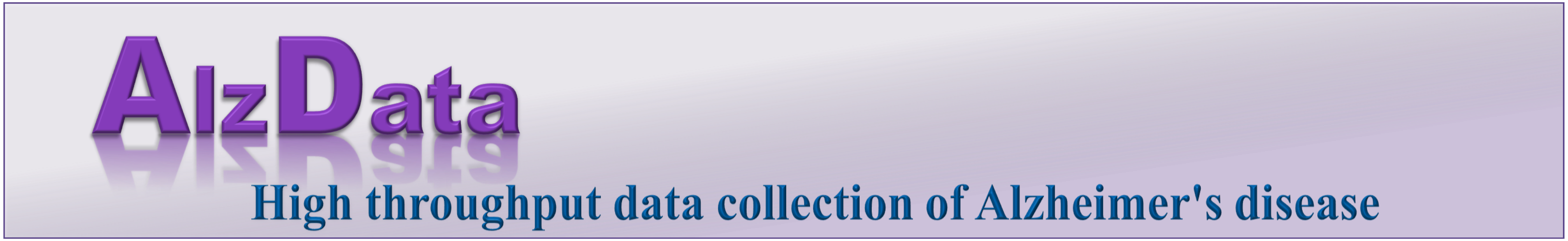

Home Differential Expression CFG Rank Single Cell Expression Chinese AD Exome Systems transcriptome More

Cross-platform nomalized expression level of *APLNR*

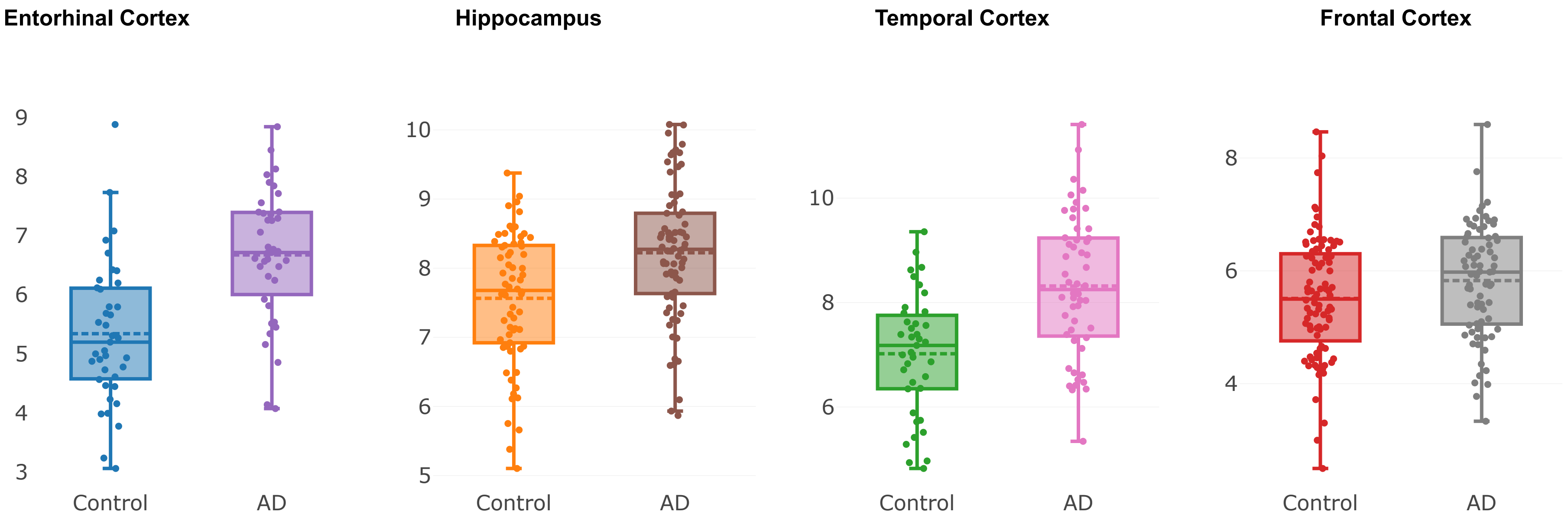

APLNR: apelin receptor			
**Brain region**	**log2 FoldChange**	**P-value**	**FDR**
Entorhinal Cortex	1.35	1.55102406555381e-06	0.001
Hippocampus	0.61	0.000274970506586381	0.01
Temporal Cortex	1.3	4.41832939908563e-06	0.000257847034554186
Frontal Cortex	0.37	0.008	0.043

Cross-platform nomalized expression level of *TGFBR3*

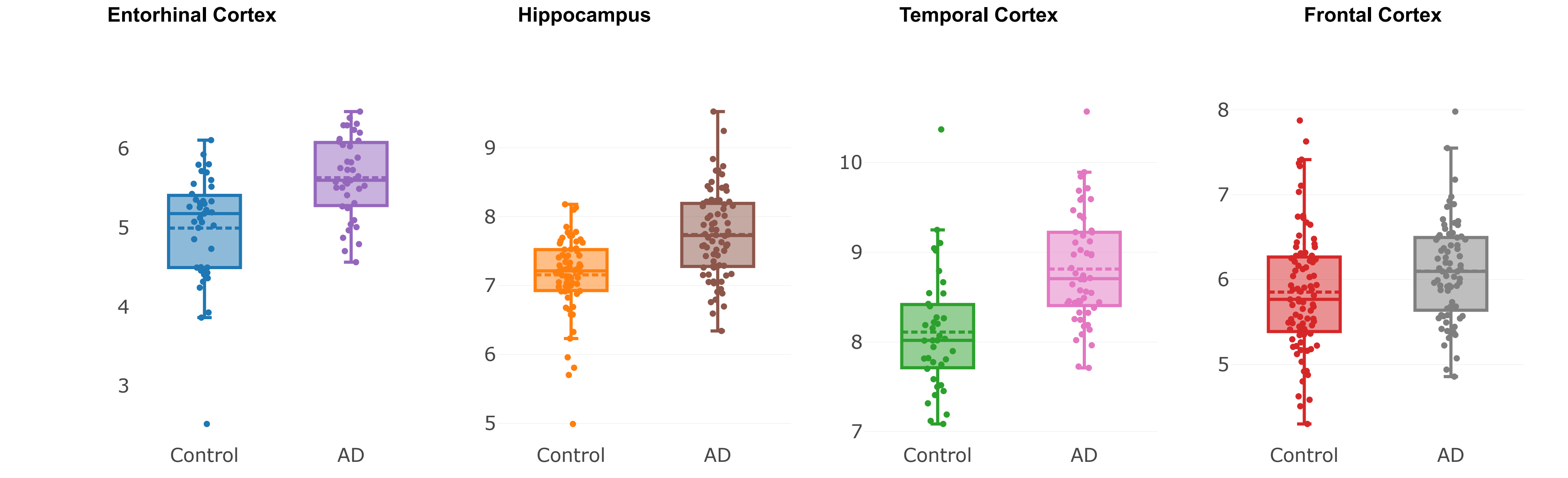

TGFBR3: transforming growth factor beta receptor III			
**Brain region**	**log2 FoldChange**	**P-value**	**FDR**
Entorhinal Cortex	0.58	5.95205321927057e-05	0.004
Hippocampus	0.58	1.52180006150554e-07	0.000248281680034629
Temporal Cortex	0.72	8.06518694047878e-07	9.74438406519565e-05
Frontal Cortex	0.37	0.000103513088125332	0.003

Cross-platform nomalized expression level of *PSMD8*

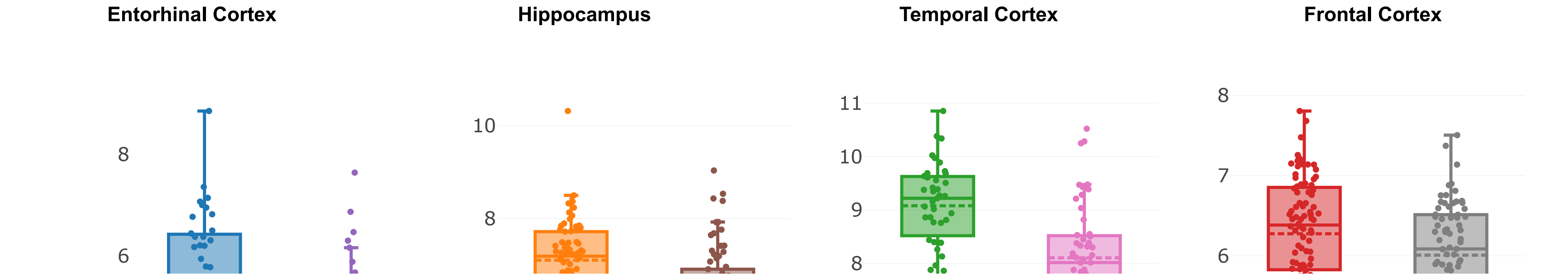

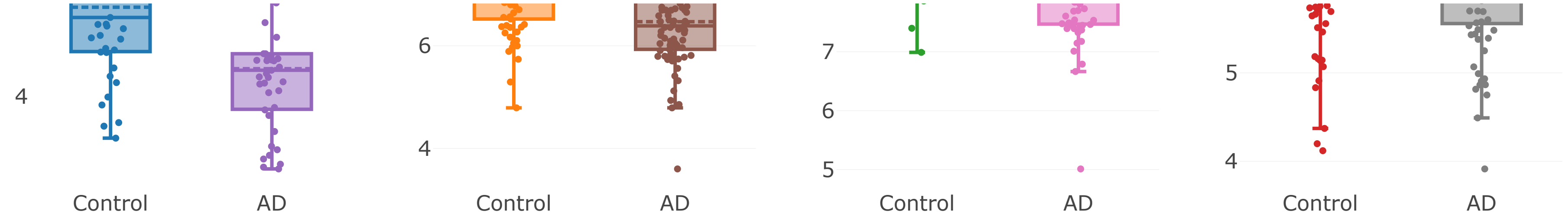

PSMD8: "proteasome 26S subunit, non-ATPase 8"			
**Brain region**	**log2 FoldChange**	**P-value**	**FDR**
Entorhinal Cortex	-1.13	7.08613428504574e-05	0.004
Hippocampus	-0.69	1.5440648169472e-05	0.002
Temporal Cortex	-0.98	2.89153482123267e-06	0.000198744826712725
Frontal Cortex	-0.22	0.006	0.035

Cross-platform nomalized expression level of *FABP3*

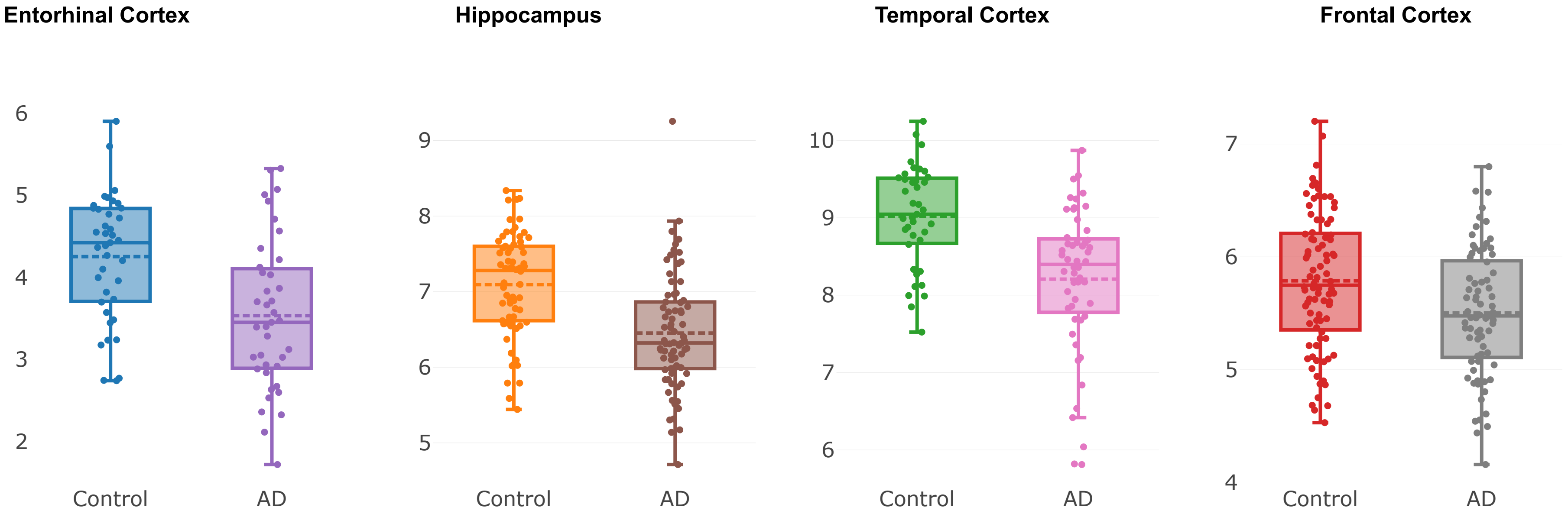

FABP3: fatty acid binding protein 3			
**Brain region**	**log2 FoldChange**	**P-value**	**FDR**
Entorhinal Cortex	-0.74	0.000218006097241711	0.007

Hippocampus	-0.61	3.35952894470111e-06	0.001
Temporal Cortex	-0.81	1.18847750804907e-05	0.000462967371838259
Frontal Cortex	-0.26	0.000382590683614315	0.006

Cross-platform nomalized expression level of *CHGB*

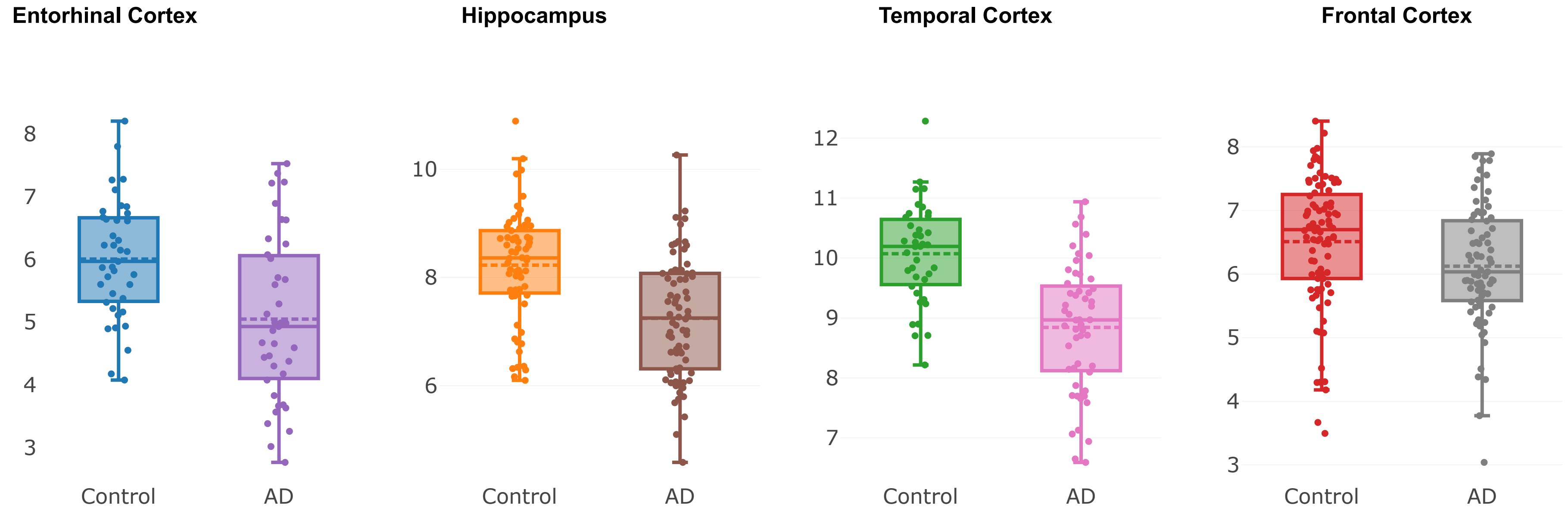

CHGB: chromogranin B			
**Brain region**	**log2 FoldChange**	**P-value**	**FDR**
Entorhinal Cortex	-1	0.000177367183036354	0.006
Hippocampus	-0.92	1.39557733340001e-06	0.001
Temporal Cortex	-1.25	1.76802834639966e-08	1.6243831713245e-05
Frontal Cortex	-0.48	0.000298806205859076	0.005

Cross-platform nomalized expression level of *CXCR4*

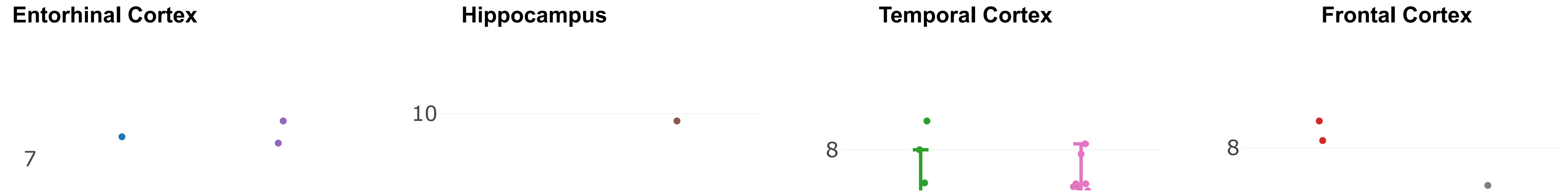

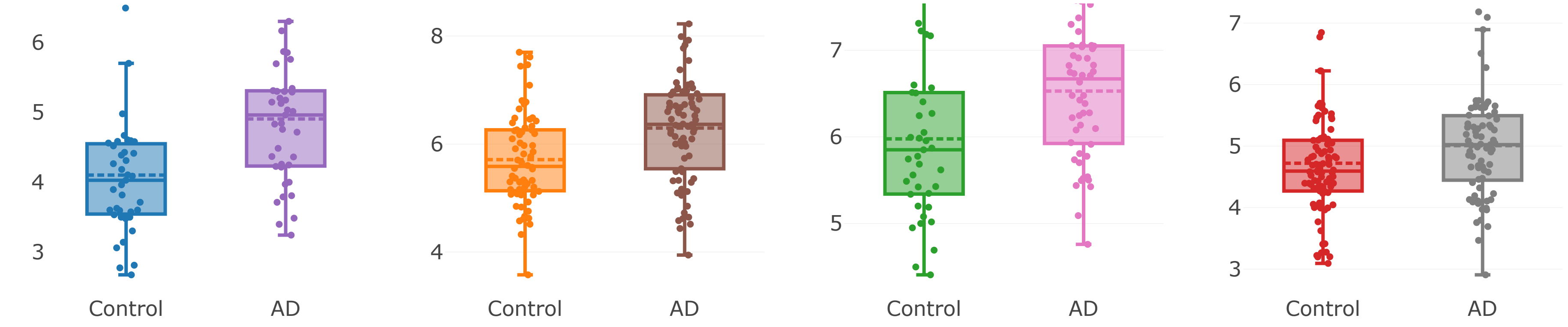

CXCR4: C-X-C motif chemokine receptor 4			
**Brain region**	**log2 FoldChange**	**P-value**	**FDR**
Entorhinal Cortex	0.8	0.00032567084854634	0.008
Hippocampus	0.56	0.001	0.017
Temporal Cortex	0.55	0.003	0.021
Frontal Cortex	0.5	3.79196451084915e-05	0.002

Cross-platform nomalized expression level of *GFAP*

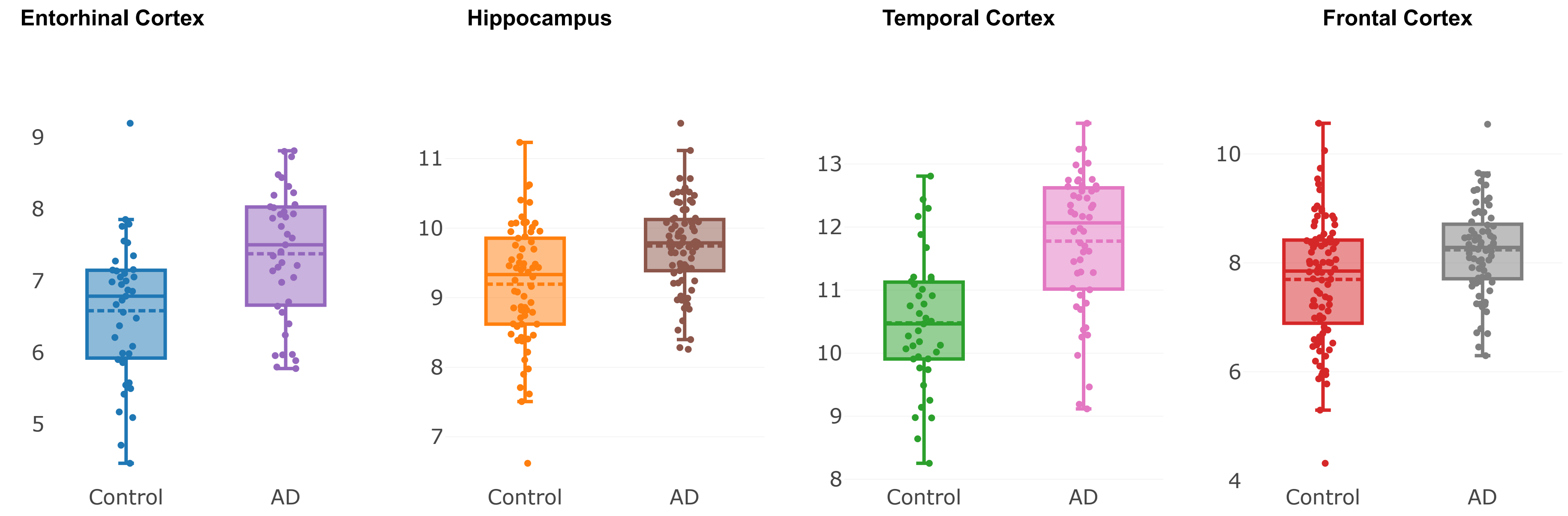

GFAP: glial fibrillary acidic protein			
**Brain region**	**log2 FoldChange**	**P-value**	**FDR**
Entorhinal Cortex	0.78	0.00039348554547264	0.009
Hippocampus	0.49	0.000209885493635492	0.008
Temporal Cortex	1.3	1.17405837683712e-07	3.61934563091175e-05
Frontal Cortex	0.61	1.14510423722581e-06	0.000226920482395707

Cross-platform nomalized expression level of *FGF12*

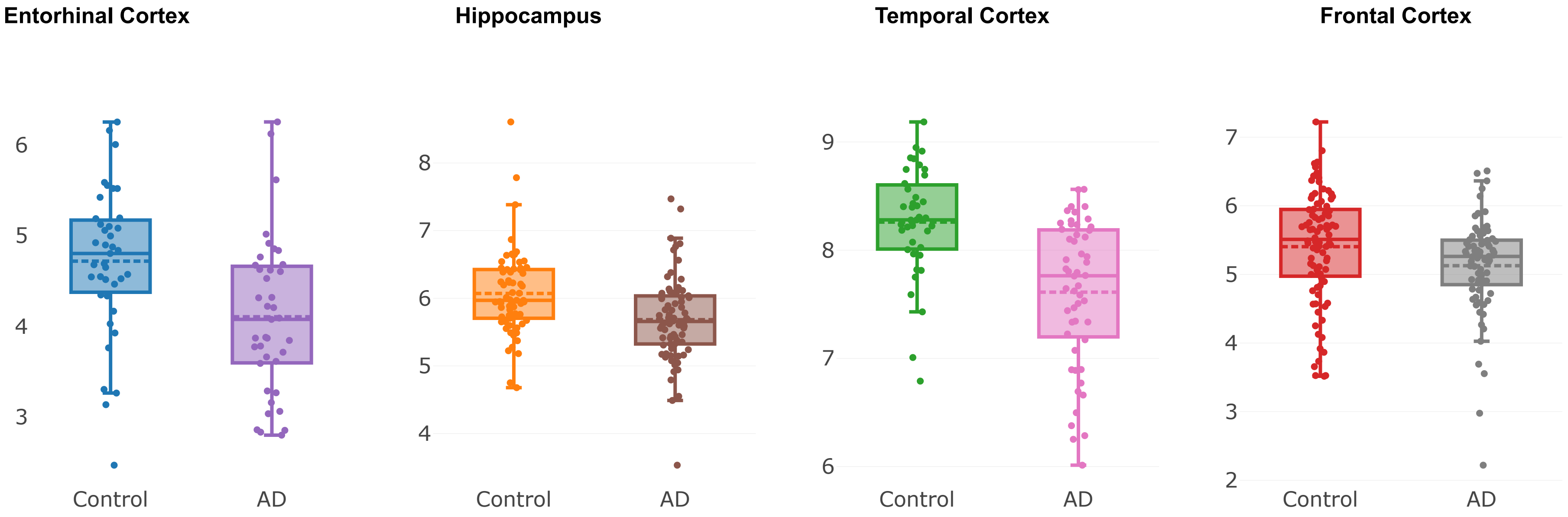

FGF12: fibroblast growth factor 12			
**Brain region**	**log2 FoldChange**	**P-value**	**FDR**
Entorhinal Cortex	-0.66	0.001	0.014
Hippocampus	-0.35	0.002	0.029
Temporal Cortex	-0.66	1.97578904717613e-06	0.00016166972282846
Frontal Cortex	-0.39	0.000485442583968057	0.007

Cross-platform nomalized expression level of *UCHL1*

Entorhinal Cortex **Hippocampus** **Temporal Cortex** **Frontal Cortex**

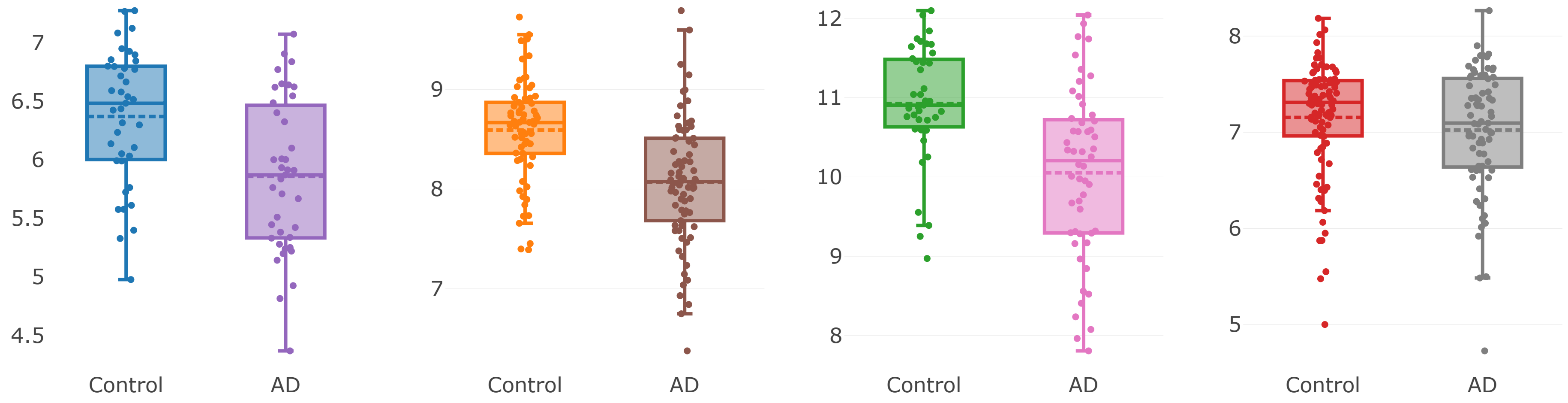

UCHL1: ubiquitin C-terminal hydrolase L1			
**Brain region**	**log2 FoldChange**	**P-value**	**FDR**
Entorhinal Cortex	-0.51	0.000489141797544756	0.01
Hippocampus	-0.48	4.6483929886917e-06	0.001
Temporal Cortex	-0.91	1.33658296967844e-05	0.000499281536861764
Frontal Cortex	-0.23	0.007	0.039

Cross-platform nomalized expression level of *COX5B*

Entorhinal Cortex **Hippocampus** **Temporal Cortex** **Frontal Cortex**

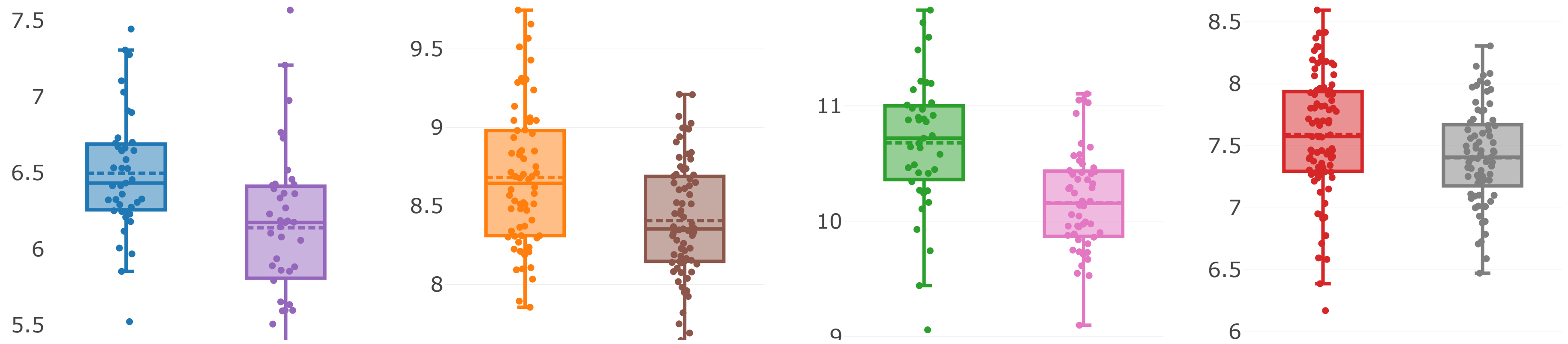

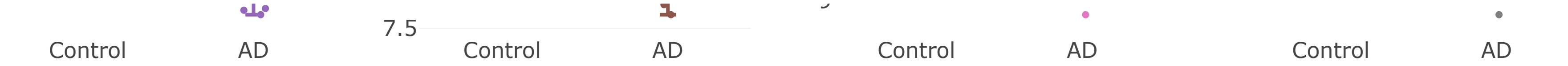

COX5B: cytochrome c oxidase subunit 5B			
**Brain region**	**log2 FoldChange**	**P-value**	**FDR**
Entorhinal Cortex	-0.41	0.00014486048091775	0.006
Hippocampus	-0.26	0.001	0.015
Temporal Cortex	-0.53	1.15746719453256e-05	0.000456232599630113
Frontal Cortex	-0.14	0.009	0.046

Cross-platform nomalized expression level of *UBE2N*

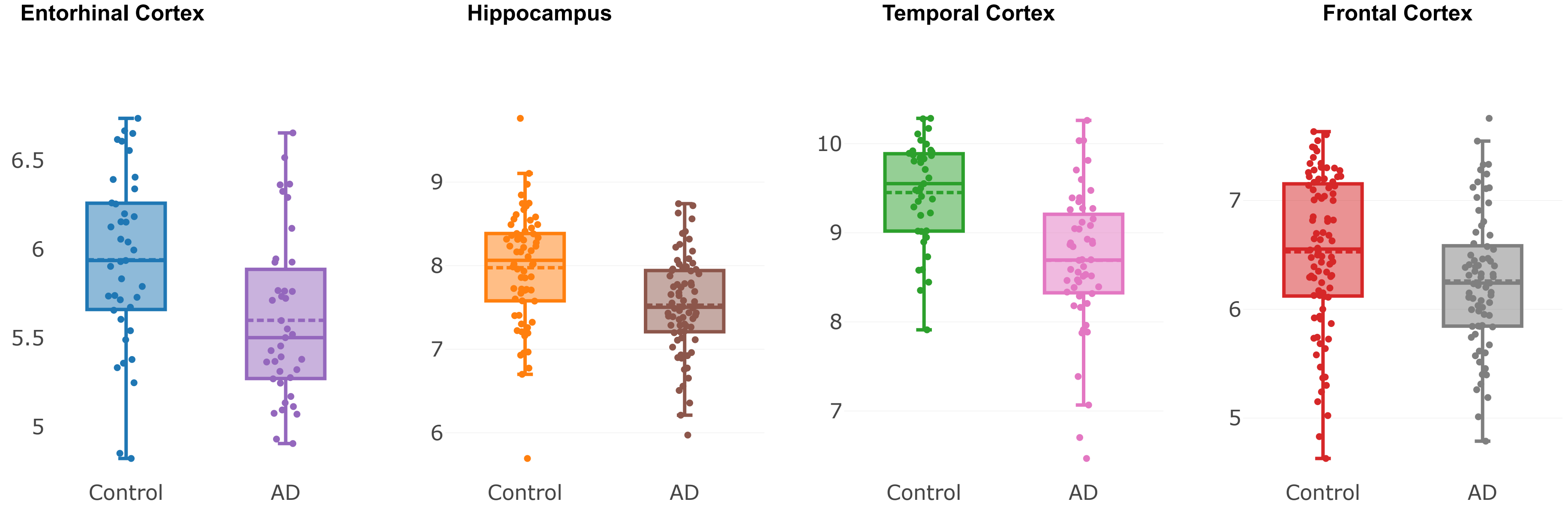

UBE2N: ubiquitin conjugating enzyme E2 N			
**Brain region**	**log2 FoldChange**	**P-value**	**FDR**
Entorhinal Cortex	-0.36	0.002	0.02
Hippocampus	-0.39	0.000386240742900048	0.012
Temporal Cortex	-0.78	7.2811263686117e-07	9.1546844951691e-05
Frontal Cortex	-0.39	1.44367975699684e-05	0.001

Cross-platform nomalized expression level of *MAP3K5*

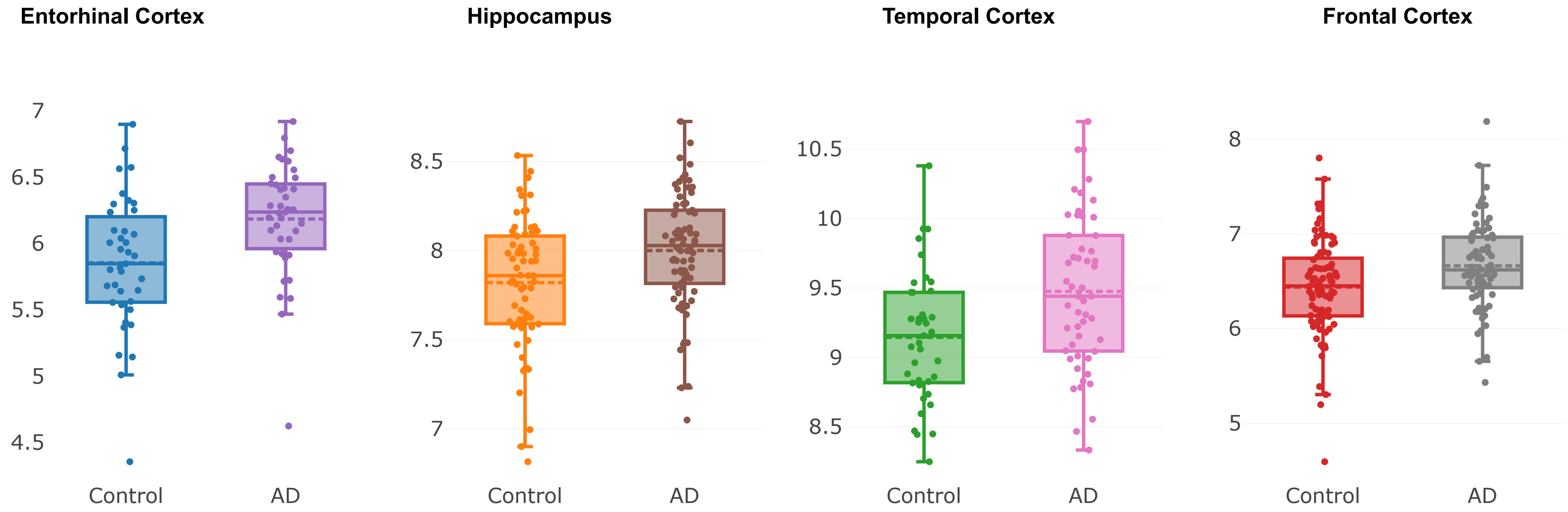

MAP3K5: mitogen-activated protein kinase kinase kinase 5			
**Brain region**	**log2 FoldChange**	**P-value**	**FDR**
Entorhinal Cortex	0.3	0.008	0.05
Hippocampus	0.19	0.003	0.04
Temporal Cortex	0.35	0.003	0.019
Frontal Cortex	0.19	0.006	0.035

Cross-platform nomalized expression level of *SYP*

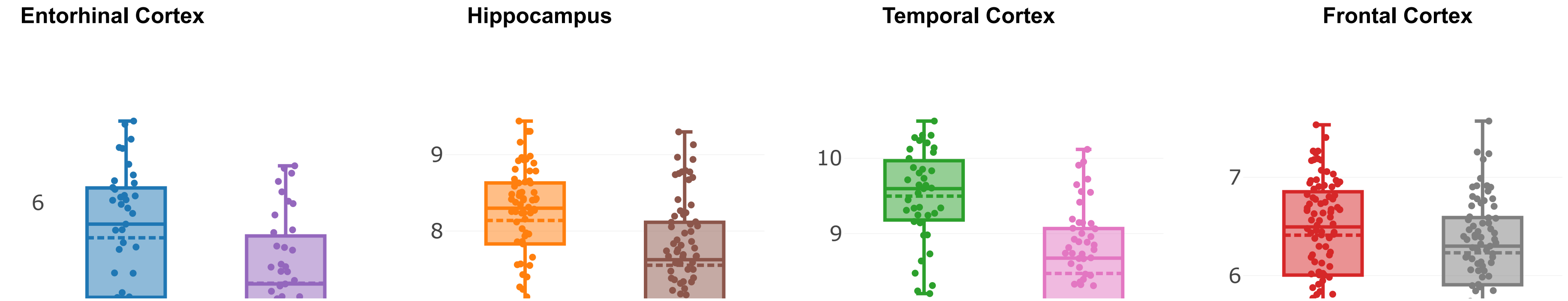

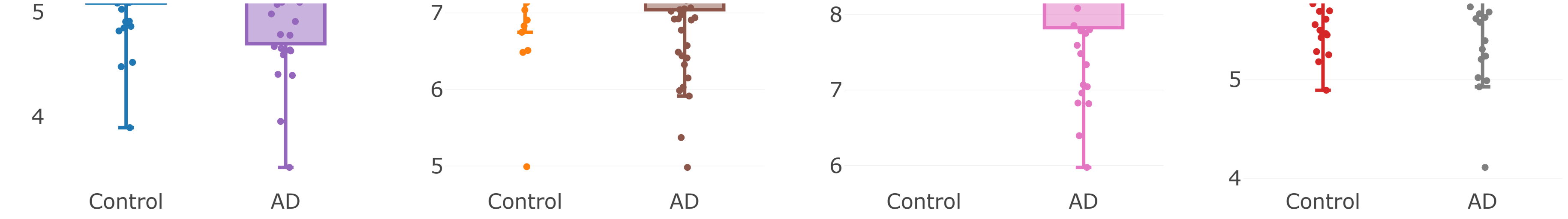

SYP: synaptophysin			
**Brain region**	**log2 FoldChange**	**P-value**	**FDR**
Entorhinal Cortex	-0.43	0.007	0.047
Hippocampus	-0.55	0.000201142983302657	0.008
Temporal Cortex	-1.05	2.73987979433049e-08	1.6243831713245e-05
Frontal Cortex	-0.27	0.001	0.011

Cross-platform nomalized expression level of *CD200*

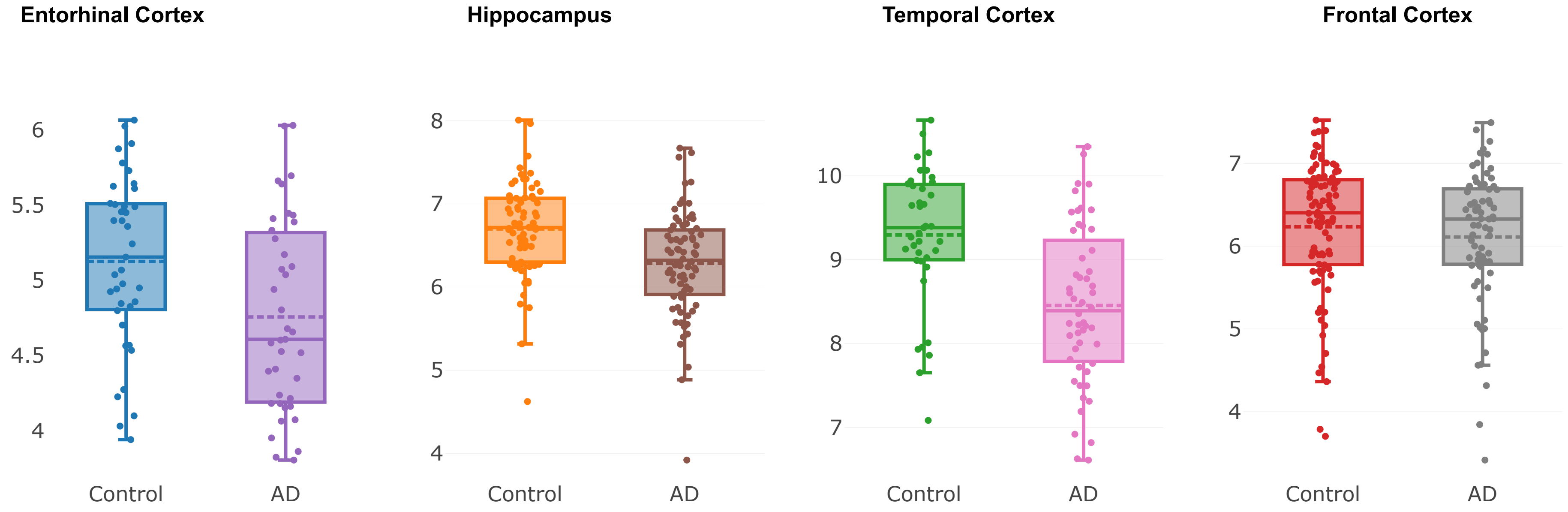

CD200: CD200 molecule			
**Brain region**	**log2 FoldChange**	**P-value**	**FDR**
Entorhinal Cortex	-0.38	0.007	0.048

Hippocampus	-0.39	0.000382428570958962	0.012
Temporal Cortex	-0.9	3.88440961223038e-06	0.000239332249614115
Frontal Cortex	-0.28	0.008	0.041

Cross-platform nomalized expression level of *GJA1*

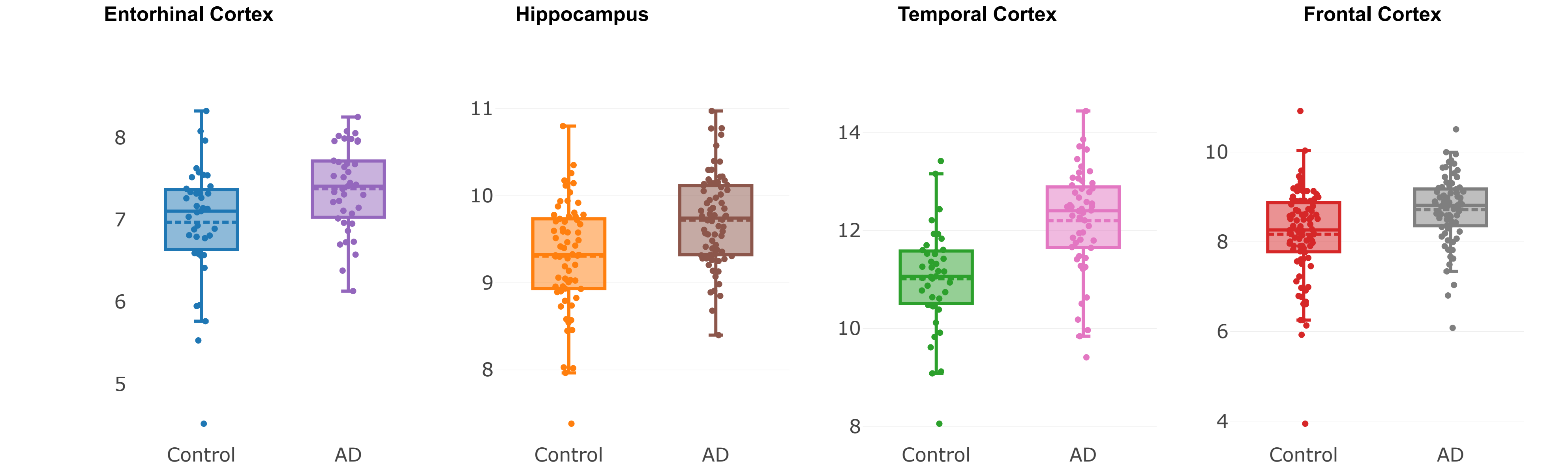

GJA1: gap junction protein alpha 1			
**Brain region**	**log2 FoldChange**	**P-value**	**FDR**
Entorhinal Cortex	0.37	0.011	0.064
Hippocampus	0.44	2.20305228369346e-05	0.002
Temporal Cortex	1.21	3.70142615854669e-07	6.4795329052601e-05
Frontal Cortex	0.44	0.001	0.009

Note:

All differential expression results were adjusted for age and sex of samples.

Dataset of Entorhinal Cortex: GSE26927, GSE26972, GSE48350, GSE5281.

Dataset of Hippocampus: GSE28146, GSE29378, GSE36980, GSE48350, GSE5281.

Dataset of Temporal Cortex: GSE29652, GSE36980, GSE37263, GSE5281.

Dataset of Frontal Cortex: GSE12685, GSE36980, GSE48350, GSE5281, GSE53890, GSE66333.

Copyright©2016-2018 Kunming Institute of Zoology, Chinese Academy of Sciences. All Rights Reserved.
