# Supplementary figures and images for: Integrating TSPO PET imaging and transcriptomics to unveil the role of neuroinflammation and amyloid-β deposition in Alzheimer’s disease
Source: Eur J Nucl Med Mol Imaging. 2023 Oct 6;51(2):455–67. doi: 10.1007/s00259-023-06446-3 (PMC10774172; doi:10.1007/s00259-023-06446-3)

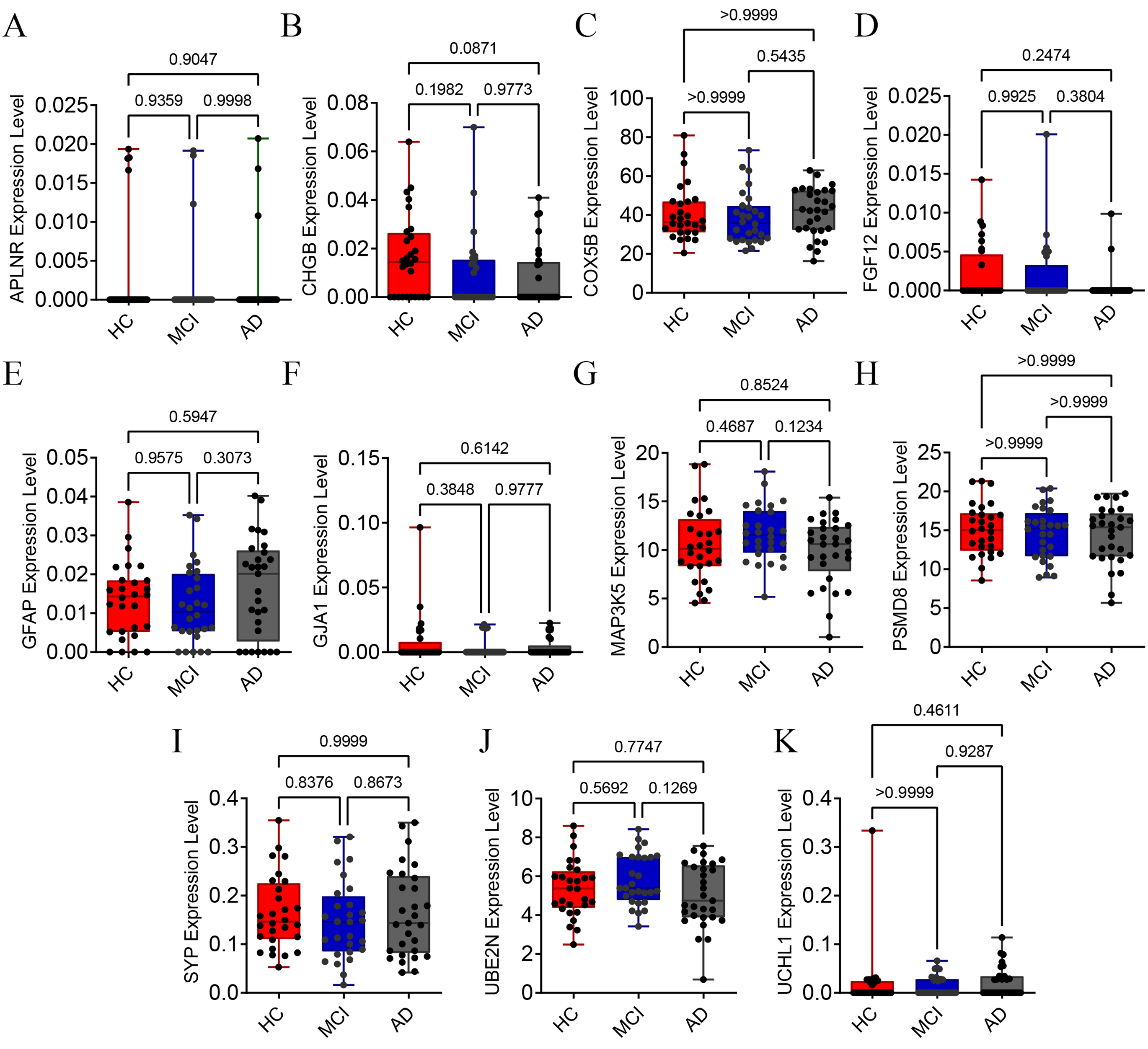

Supplement: Supplementary file 3 — Supplementary file3 (JPG 1479 KB) [file 259_2023_6446_MOESM3_ESM.jpg]

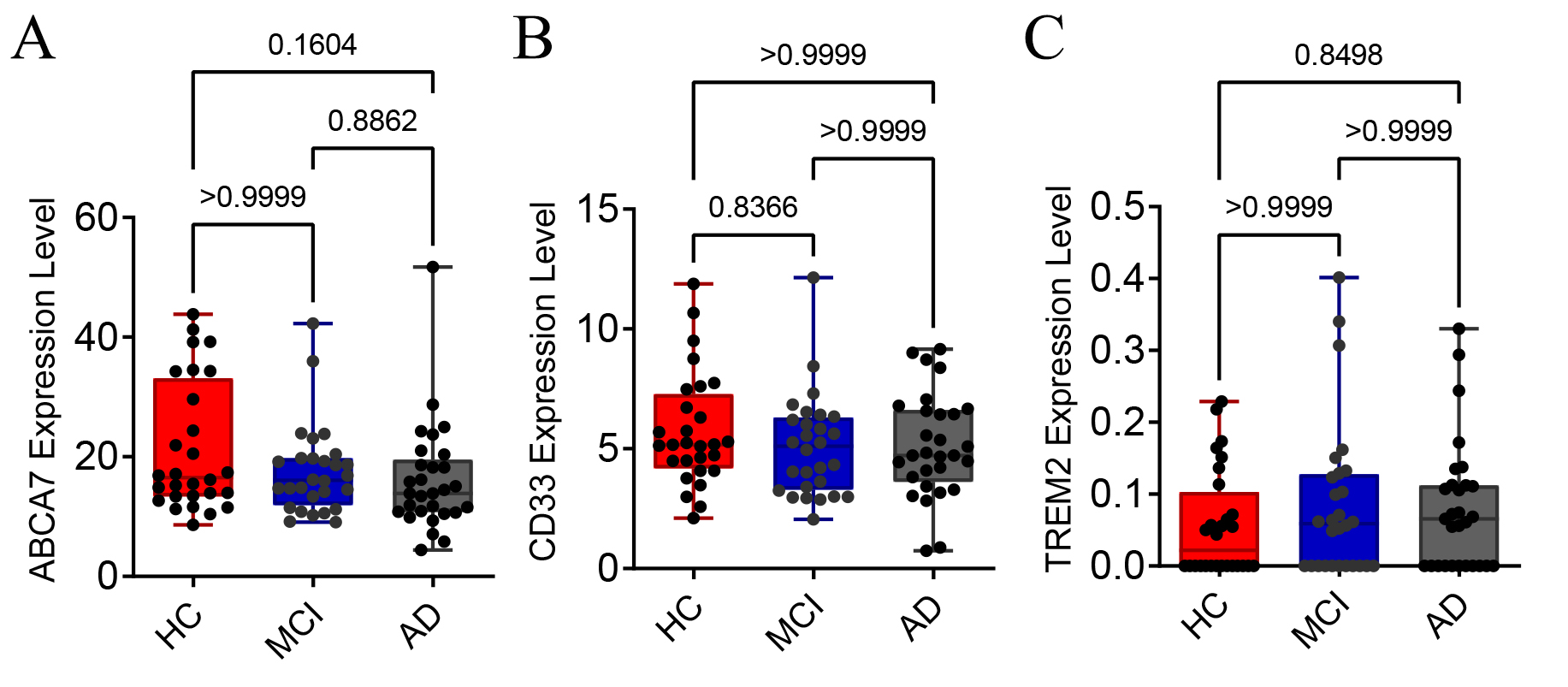

Supplement: Supplementary file 4 — Supplementary file4 (JPG 376 KB) [file 259_2023_6446_MOESM4_ESM.jpg]

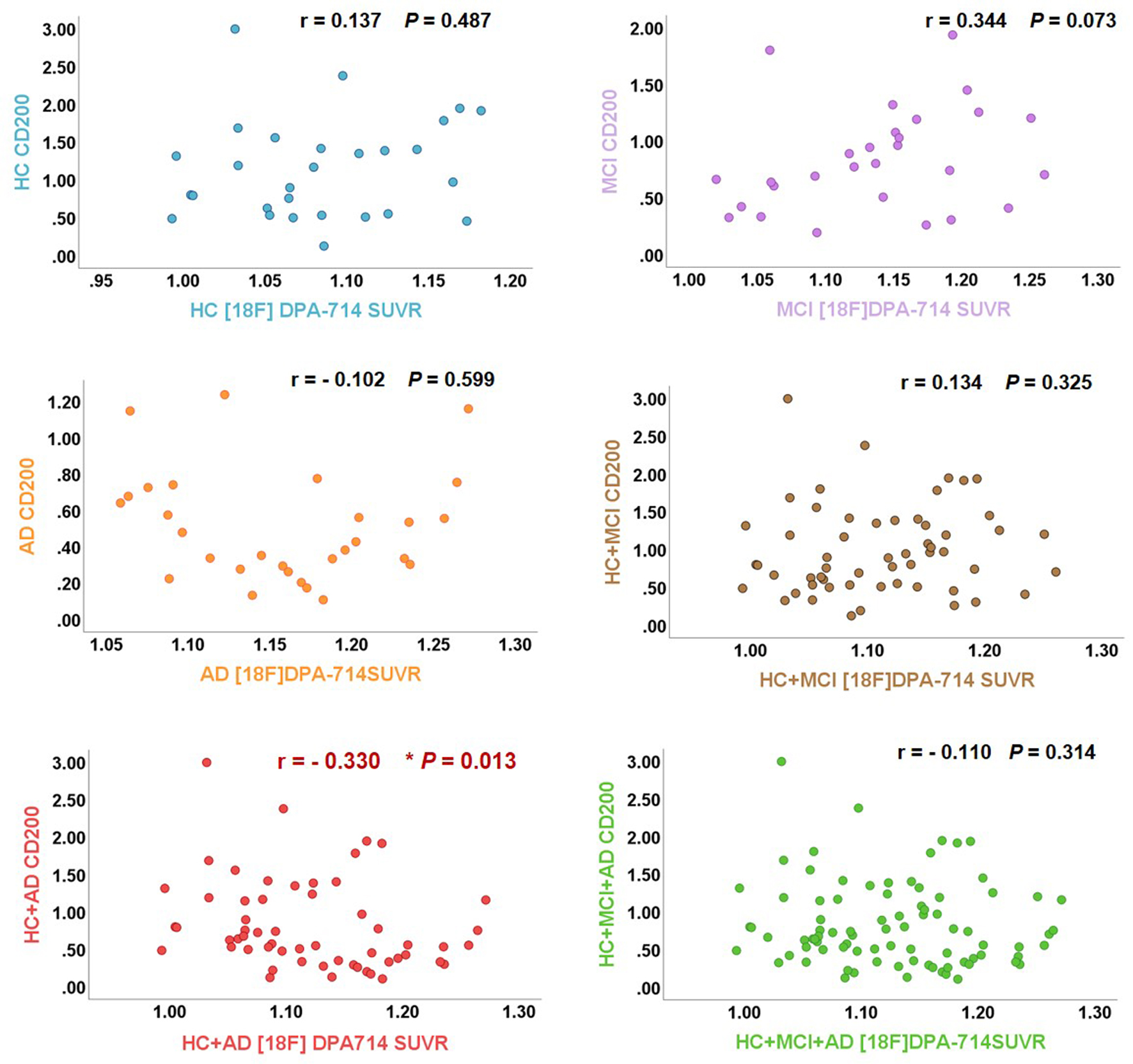

Supplement: Supplementary file 5 — Supplementary file5 (JPG 760 KB) [file 259_2023_6446_MOESM5_ESM.jpg]

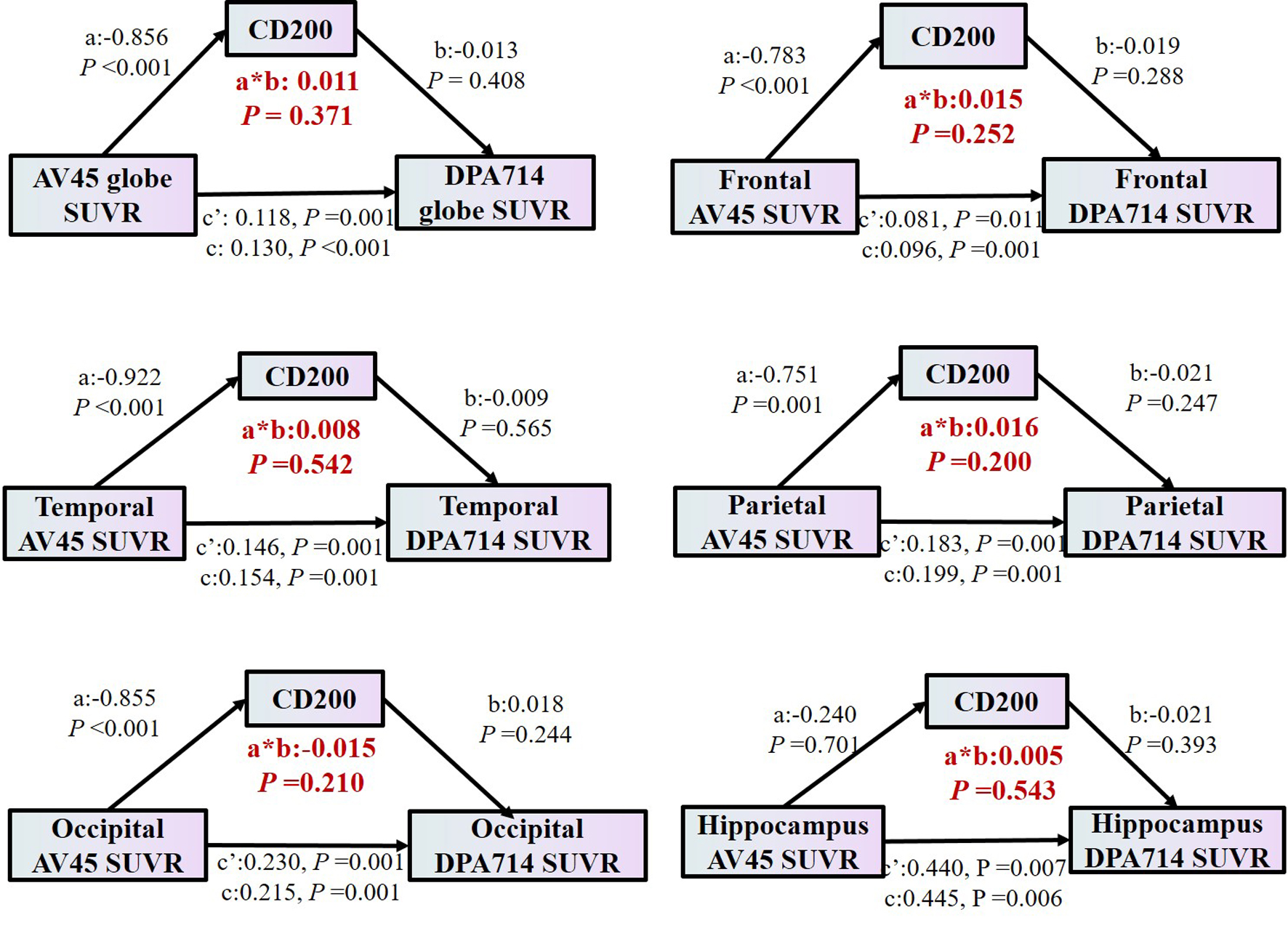

Supplement: Supplementary file 6 — Supplementary file6 (JPG 1028 KB) [file 259_2023_6446_MOESM6_ESM.jpg]
