# Supplementary material for: Integrating TSPO PET imaging and transcriptomics to unveil the role of neuroinflammation and amyloid-β deposition in Alzheimer’s disease
Source: Eur J Nucl Med Mol Imaging. 2023 Oct 6;51(2):455–67. doi: 10.1007/s00259-023-06446-3 (PMC10774172; doi:10.1007/s00259-023-06446-3)
Supplement: Supplementary file 7 — Supplementary file7 (DOCX 31 KB) [file 259_2023_6446_MOESM7_ESM.docx]

**Table S1 TSPO genotypers rs6971 polymorphism test results of all participants.**

| Sample | Gene | SNP | Reference base | Result | Mutation type |
| --- | --- | --- | --- | --- | --- |
| D1 | TSPO | rs6971 | A | GG | Homozygous mutation |
| D2 | TSPO | rs6971 | A | GG | Homozygous mutation |
| D3 | TSPO | rs6971 | A | GG | Homozygous mutation |
| D4 | TSPO | rs6971 | A | GG | Homozygous mutation |
| D5 | TSPO | rs6971 | A | GG | Homozygous mutation |
| D6 | TSPO | rs6971 | A | GG | Homozygous mutation |
| D7 | TSPO | rs6971 | A | GG | Homozygous mutation |
| D8 | TSPO | rs6971 | A | GG | Homozygous mutation |
| D9 | TSPO | rs6971 | A | GG | Homozygous mutation |
| D10 | TSPO | rs6971 | A | GG | Homozygous mutation |
| D11 | TSPO | rs6971 | A | GG | Homozygous mutation |
| D12 | TSPO | rs6971 | A | GG | Homozygous mutation |
| D13 | TSPO | rs6971 | A | GG | Homozygous mutation |
| D14 | TSPO | rs6971 | A | GG | Homozygous mutation |
| D15 | TSPO | rs6971 | A | GG | Homozygous mutation |
| D16 | TSPO | rs6971 | A | GG | Homozygous mutation |
| D17 | TSPO | rs6971 | A | GG | Homozygous mutation |
| D18 | TSPO | rs6971 | A | GG | Homozygous mutation |
| D19 | TSPO | rs6971 | A | GG | Homozygous mutation |
| D20 | TSPO | rs6971 | A | GG | Homozygous mutation |
| D21 | TSPO | rs6971 | A | GG | Homozygous mutation |
| D22 | TSPO | rs6971 | A | GG | Homozygous mutation |
| D23 | TSPO | rs6971 | A | GG | Homozygous mutation |
| D24 | TSPO | rs6971 | A | GG | Homozygous mutation |
| D25 | TSPO | rs6971 | A | GG | Homozygous mutation |
| D26 | TSPO | rs6971 | A | GG | Homozygous mutation |
| D27 | TSPO | rs6971 | A | GG | Homozygous mutation |
| D28 | TSPO | rs6971 | A | GG | Homozygous mutation |
| D29 | TSPO | rs6971 | A | GG | Homozygous mutation |
| D30 | TSPO | rs6971 | A | GG | Homozygous mutation |
| D31 | TSPO | rs6971 | A | GG | Homozygous mutation |
| D32 | TSPO | rs6971 | A | GG | Homozygous mutation |
| D33 | TSPO | rs6971 | A | GG | Homozygous mutation |
| D34 | TSPO | rs6971 | A | GG | Homozygous mutation |
| D35 | TSPO | rs6971 | A | GG | Homozygous mutation |
| D36 | TSPO | rs6971 | A | GG | Homozygous mutation |
| D37 | TSPO | rs6971 | A | GG | Homozygous mutation |
| D38 | TSPO | rs6971 | A | GG | Homozygous mutation |
| D39 | TSPO | rs6971 | A | GG | Homozygous mutation |
| D40 | TSPO | rs6971 | A | GG | Homozygous mutation |
| D41 | TSPO | rs6971 | A | GG | Homozygous mutation |
| D42 | TSPO | rs6971 | A | GG | Homozygous mutation |
| D43 | TSPO | rs6971 | A | GG | Homozygous mutation |
| D44 | TSPO | rs6971 | A | GG | Homozygous mutation |
| D45 | TSPO | rs6971 | A | GG | Homozygous mutation |
| D46 | TSPO | rs6971 | A | GG | Homozygous mutation |
| D47 | TSPO | rs6971 | A | GG | Homozygous mutation |
| D48 | TSPO | rs6971 | A | GG | Homozygous mutation |
| D49 | TSPO | rs6971 | A | GG | Homozygous mutation |
| D50 | TSPO | rs6971 | A | GG | Homozygous mutation |
| D51 | TSPO | rs6971 | A | GG | Homozygous mutation |
| D52 | TSPO | rs6971 | A | GG | Homozygous mutation |
| D53 | TSPO | rs6971 | A | GG | Homozygous mutation |
| D54 | TSPO | rs6971 | A | GG | Homozygous mutation |
| D55 | TSPO | rs6971 | A | GG | Homozygous mutation |
| D56 | TSPO | rs6971 | A | GG | Homozygous mutation |
| D57 | TSPO | rs6971 | A | GG | Homozygous mutation |
| D58 | TSPO | rs6971 | A | GG | Homozygous mutation |
| D59 | TSPO | rs6971 | A | GG | Homozygous mutation |
| D60 | TSPO | rs6971 | A | GG | Homozygous mutation |
| D61 | TSPO | rs6971 | A | GG | Homozygous mutation |
| D62 | TSPO | rs6971 | A | GG | Homozygous mutation |
| D63 | TSPO | rs6971 | A | GG | Homozygous mutation |
| D64 | TSPO | rs6971 | A | GG | Homozygous mutation |
| D65 | TSPO | rs6971 | A | GG | Homozygous mutation |
| D66 | TSPO | rs6971 | A | GG | Homozygous mutation |
| D67 | TSPO | rs6971 | A | GG | Homozygous mutation |
| D68 | TSPO | rs6971 | A | GG | Homozygous mutation |
| D69 | TSPO | rs6971 | A | GG | Homozygous mutation |
| D70 | TSPO | rs6971 | A | GG | Homozygous mutation |
| D71 | TSPO | rs6971 | A | GG | Homozygous mutation |
| D72 | TSPO | rs6971 | A | GG | Homozygous mutation |
| D73 | TSPO | rs6971 | A | GG | Homozygous mutation |
| D74 | TSPO | rs6971 | A | GG | Homozygous mutation |
| D75 | TSPO | rs6971 | A | GG | Homozygous mutation |
| D76 | TSPO | rs6971 | A | GG | Homozygous mutation |
| D77 | TSPO | rs6971 | A | GG | Homozygous mutation |
| D78 | TSPO | rs6971 | A | GG | Homozygous mutation |
| D79 | TSPO | rs6971 | A | GG | Homozygous mutation |
| D80 | TSPO | rs6971 | A | GG | Homozygous mutation |
| D81 | TSPO | rs6971 | A | GG | Homozygous mutation |
| D82 | TSPO | rs6971 | A | GG | Homozygous mutation |
| D83 | TSPO | rs6971 | A | GG | Homozygous mutation |
| D84 | TSPO | rs6971 | A | GG | Homozygous mutation |
| D85 | TSPO | rs6971 | A | GG | Homozygous mutation |

**Table S2 [^18^F] DPA-714 PET SUVR and ANCOVA analysis between controls, MCI and AD three groups, ANCOVA was performed with age, sex and education as covariates.**

|  | Controls | | MCI | | AD | | *P* value | | |
| --- | --- | --- | --- | --- | --- | --- | --- | --- | --- |
|  | Mean SUVR | SD | Mean SUVR | SD | Mean SUVR | SD | MCI*vs.*HC | AD *vs.*HC | MCI *vs.*AD |
| Globe | 1.0839 | 0.05626 | 1.1375 | 0.06793 | 1.1582 | 0.06444 | 0.0003 | <0.0001 | 0.5325 |
| Frontal | 1.082 | 0.07007 | 1.1347 | 0.07427 | 1.1512 | 0.08786 | 0.0071 | 0.0027 | 0.7813 |
| Parietal | 1.0504 | 0.06862 | 1.1179 | 0.08961 | 1.1579 | 0.0835 | 0.0003 | <0.0001 | 0.2236 |
| Occipital | 1.1305 | 0.07847 | 1.1697 | 0.07073 | 1.1749 | 0.07801 | 0.006 | 0.0177 | 0.6466 |
| Temporal | 0.896 | 0.05368 | 0.9557 | 0.06982 | 0.9854 | 0.07525 | 0.0001 | <0.0001 | 0.2552 |
| Hippocampus | 1.1276 | 0.9757 | 1.1632 | 0.08018 | 1.1732 | 0.09404 | 0.0336 | 0.0283 | 0.9748 |
| Parahippocampal | 0.984 | 0.05299 | 1.0193 | 0.5862 | 1.0427 | 0.06184 | 0.0114 | 0.0003 | 0.2322 |
| Anteriorcingulate, ACC | 1.1793 | 0.08445 | 1.2155 | 0.0956 | 1.193 | 0.09101 | 0.1757 | 0.6787 | 0.324 |
| Postcingulate, PCC | 1.1311 | 0.07263 | 1.1929 | 0.08826 | 1.232 | 0.08742 | 0.0035 | <0.0001 | 0.1977 |
| Precuneus | 1.0827 | 0.07429 | 1.1651 | 0.11402 | 1.2095 | 0.0829 | 0.0005 | <0.0002 | 0.1763 |
| Entorhinal cortex | 1.0304 | 0.6293 | 1.0606 | 0.06816 | 1.0789 | 0.07494 | 0.036 | 0.0047 | 0.4557 |
| Thalamus | 1.3659 | 0.0949 | 1.4203 | 0.11323 | 1.4057 | 0.11807 | 0.0835 | 0.195 | 0.6461 |
| Caudate | 0.8236 | 0.5904 | 0.8262 | 0.06482 | 0.8064 | 0.08447 | 0.4506 | 0.4877 | 0.1391 |
| Putamen | 1.0468 | 0.08077 | 1.1092 | 0.09011 | 1.1053 | 0.10427 | 0.0279 | 0.0247 | 0.993 |
| Striatum | 0.9515 | 0.05419 | 0.9851 | 0.06411 | 0.9762 | 0.01738 | 0.0853 | 0.2247 | 0.5764 |
| Amygdala | 1.2237 | 0.09282 | 1.3229 | 0.12312 | 1.3828 | 0.09287 | 0.0004 | <0.0001 | 0.023 |
| Whitematter | 1.0607 | 0.0793 | 1.1032 | 0.065 | 1.1384 | 0.07311 | 0.0365 | 0.0004 | 0.1177 |
|  |  |  |  |  |  |  |  |  |  |

**Table S3: 15 immune-related differentially expressed genes between Alzheimer’s disease and healthy controls.**

| Gene | Entorhinal Cortex | | | | Hippocampus | | | | Temporal Cortex | | | | Frontal Cortex | | |
| --- | --- | --- | --- | --- | --- | --- | --- | --- | --- | --- | --- | --- | --- | --- | --- |
|  | log2 FC | P-value | FDR | log2 FC | | P-value | FDR | log2 FC | | P-value | FDR | log2 FC | | P-value | FDR |
| APLNR | 1.35 | 1.55e-06 | 0.001 | 0.61 | | 2.75e-04 | 0.01 | 1.3 | | 4.42-06 | 2.58e-04 | 0.37 | | 0.008 | 0.043 |
| TGFBR3 | 0.58 | 5.95e-05 | 0.004 | 0.58 | | 1.52e-07 | 2.48e-04 | 0.72 | | 8.07e-07 | 9.74e-05 | 0.37 | | 1.03e-4 | 0.003 |
| PSMD8 | -1.13 | 7.09e-05 | 0.004 | -0.69 | | 1.54e-05 | 0.002 | -0.98 | | 2.89e-06 | 1.99e-04 | -0.22 | | 0.006 | 0.035 |
| FABP3 | -0.74 | 2.18e-04 | 0.007 | -0.61 | | 3.36e-06 | 0.001 | -0.81 | | 1.19e-05 | 4.63e-04 | -0.26 | | 3.83e-04 | 0.006 |
| CHGB | -1 | 1.77e-04 | 0.006 | -0.92 | | 1.40e-06 | 0.001 | -1.25 | | 1.77e-08 | 1.62e-05 | 0.48 | | 2.99e-04 | 0.005 |
| CXCR4 | 0.8 | 3.26e-4 | 0.008 | 0.56 | | 0.001 | 0.017 | 0.55 | | 0.003 | 0.021 | 0.5 | | 3.79e-05 | 0.002 |
| GFAP | 0.78 | 3.93e-04 | 0.009 | 0.49 | | 2.10e-04 | 0.008 | 1.3 | | 1.17e-07 | 3.62e-05 | 0.61 | | 1.15e-06 | 2.27e-04 |
| FGF12 | -0.66 | 0.001 | 0.014 | -0.35 | | 0.002 | 0.029 | -0.66 | | 1.98e-06 | 1.62e-04 | -0.39 | | 4.85e-04 | 0.007 |
| UCHL1 | -0.51 | 4.89e-04 | 0.01 | -0.48 | | 4.64e-06 | 0.001 | -0.91 | | 1.34e-05 | 5.00e-04 | -0.23 | | 0.007 | 0.039 |
| COX5B | -0.41 | 1.45e-04 | 0.006 | -0.26 | | 0.001 | 0.015 | 0.53 | | 1.16e-05 | 4.56e-04 | -0.14 | | 0.009 | 0.046 |
| UBE2N | -0.36 | 0.002 | 0.02 | -0.39 | | 3.86e-04 | 0.012 | -0.78 | | 7.28e-07 | 9.15e-05 | 1.44e-05 | | 1.44e-05 | 0.001 |
| MAP3K5 | 0.3 | 0.008 | 0.05 | 0.19 | | 0.003 | 0.04 | 0.35 | | 0.003 | 0.019 | 0.19 | | 0.006 | 0.035 |
| SYP | -0.43 | 0.007 | 0.047 | -0.55 | | 2.01e-4 | 0.008 | 1.05 | | 2.74e-08 | 1.62e-05 | -0.27 | | 0.001 | 0.011 |
| CD200 | -0.38 | 0.007 | 0.048 | -0.39 | | 3.82e-04 | 0.012 | -0.9 | | 3.88e-06 | 2.39e-04 | 0.28 | | 0.008 | 0.041 |
| GJA1 | 0.37 | 0.011 | 0.064 | 0.44 | | 2.20e-05 | 0.002 | 1.21 | | 3.70e-07 | 6.48e-05 | 0.44 | | 0.001 | 0.009 |

**Table S4 The Correlation of blood CD200, CXCR4, TGFGR, FABR3 level and [^18^F]DPA714 PET SUVR in HC and AD patients.**

|  | *CD200* | | *CXCR4* | | *TGFBR 3* | | *FABR3* | |
| --- | --- | --- | --- | --- | --- | --- | --- | --- |
|  | ***r***值 | ***P*** 值 | ***r***值 | ***P*** 值 | ***r***值 | ***P*** 值 | ***r***值 | ***P*** 值 |
| Globe | -0.33 | 0.013 | -0.08 | 0.55 | -0.1 | 0.467 | 0.05 | 0.724 |
| Frontal | -0.34 | 0.01 | -0.25 | 0.058 | -0.11 | 0.417 | 0.01 | 0.912 |
| Parietal | -0.32 | 0.017 | -0.11 | 0.417 | -0.17 | 0.214 | 0.007 | 0.955 |
| Occipital | -0.08 | 0.575 | 0.18 | 0.182 | -0.08 | 0.575 | 0.1 | 0.437 |
| Temporal | -0.26 | 0.047 | -0.07 | 0.617 | -0.01 | 0.933 | 0.06 | 0.661 |
| Hippocampus | -0.21 | 0.11 | 0.11 | 0.407 | 0.003 | 0.982 | 0.19 | 0.166 |
| Parahippocampal | -0.29 | 0.03 | -0.03 | 0.851 | -0.05 | -0.692 | 0.06 | 0.676 |
| Anteriorcingulate, ACC | -0.12 | 0.389 | -0.11 | 0.407 | 0.07 | 0.616 | 0.1 | 0.468 |
| Postcingulate, PCC | -0.34 | 0.009 | -0.17 | 0.2 | -0.08 | 0.575 | -0.009 | 0.955 |
| Precuneus | -0.39 | 0.003 | -0.2 | 0.129 | -0.22 | 0.107 | -0.06 | 0.661 |
| Entorhinal cortex | 0.03 | 0.813 | 0.18 | 0.186 | -0.03 | 0.813 | 0.08 | 0.55 |
| Thalamus | -0.27 | 0.045 | -0.1 | 0.467 | 0.02 | 0.871 | 0.0028 | 0.983 |
| Caudate | -0.05 | 0.741 | -0.03 | 0.813 | -0.0062 | 0.955 | -0.01 | 0.933 |
| Putamen | -0.39 | 0.002 | -0.16 | 0.223 | -0.08 | 0.562 | -0.06 | 0.676 |
| Striatum | -0.29 | 0.029 | -0.14 | 0.309 | -0.04 | 0.759 | -0.04 | 0.741 |
| Amygdala | -0.41 | 0.001 | -0.19 | 0.165 | -0.06 | 0.66 | 0.0092 | 0.955 |
| White matter | -0.34 | 0.011 | -0.0067 | 0.955 | -0.07 | 0.602 | 0.03 | 0.851 |

**Table S5 The Correlation of blood CD200 expression and [^18^F]DPA714 PET SUVR in HC , MCI and AD patients.**

|  | *CD200* | | *CXCR4* | | *TGFBR 3* | | *FABR3* | |
| --- | --- | --- | --- | --- | --- | --- | --- | --- |
|  | ***r***值 | ***P*** 值 | ***r***值 | ***P*** 值 | ***r***值 | ***P*** 值 | ***r***值 | ***P*** 值 |
| Globe | -0.110 | 0.316 | -0.080 | 0.457 | -0.030 | 0.794 | 0.02 | 0.891 |
| Frontal | -0.120 | 0.282 | -0.210 | 0.057 | -0.030 | 0.794 | 0.02 | 0.831 |
| Parietal | -0.100 | 0.339 | -0.120 | 0.288 | -0.100 | 0.372 | -0.03 | 0.759 |
| Occipital | 0.060 | 0.617 | 0.140 | 0.209 | -0.040 | 0.692 | 0.06 | 0.575 |
| Temporal | -0.070 | 0.550 | -0.010 | 0.891 | 0.040 | 0.741 | 0.02 | 0.851 |
| Hippocampus | -0.150 | 0.162 | 0.100 | 0.355 | 0.080 | 0.468 | 0.1 | 0.347 |
| Parahippocampal | -0.100 | 0.355 | 0.060 | 0.575 | 0.040 | 0.724 | 0.03 | 0.776 |
| Anteriorcingulate, ACC | 0.000 | 0.997 | -0.140 | 0.191 | 0.080 | 0.447 | 0.11 | 0.324 |
| Postcingulate, PCC | -0.120 | 0.257 | -0.150 | 0.158 | -0.040 | 0.692 | -0.03 | 0.812 |
| Precuneus | -0.160 | 0.155 | -0.190 | 0.081 | -0.120 | 0.257 | -0.05 | 0.617 |
| Entorhinal cortex | 0.050 | 0.647 | 0.200 | 0.068 | 0.040 | 0.708 | 0.1 | 0.38 |
| Thalamus | -0.100 | 0.355 | -0.090 | 0.398 | 0.090 | 0.436 | -0.02 | 0.871 |
| Caudate | 0.050 | 0.661 | -0.040 | 0.724 | 0.040 | 0.692 | 0.02 | 0.851 |
| Putamen | -0.230 | 0.033 | -0.180 | 0.091 | 0.003 | 0.982 | -0.0086 | 0.933 |
| Striatum | -0.160 | 0.135 | -0.160 | 0.135 | 0.030 | 0.794 | -0.0082 | 0.933 |
| Amygdala | -0.330 | 0.002 | -0.230 | 0.035 | -0.040 | 0.759 | -0.07 | 0.537 |
| White matter | -0.180 | 0.010 | -0.080 | 0.447 | -0.090 | 0.437 | -0.08 | 0.479 |
